# Activation of p53 signaling and regression of breast and prostate carcinoma cells by spirooxindole-benzimidazole small molecules

**DOI:** 10.3389/fphar.2024.1358089

**Published:** 2024-04-08

**Authors:** Assem Barakat, Saeed Alshahrani, Abdullah Mohammed Al-Majid, Abdullah Saleh Alamary, Matti Haukka, Marwa M. Abu-Serie, Alexander Dömling, Luis R. Domingo, Yaseen A. M. M. Elshaier

**Affiliations:** ^1^ Department of Chemistry, College of Science, King Saud University, Riyadh, Saudi Arabia; ^2^ Department of Chemistry, University of Jyväskylä, Jyväskylä, Finland; ^3^ Medical Biotechnology Department, Genetic Engineering and Biotechnology Research Institute, City of Scientific Research and Technological Applications (SRTA City), Alexandria, Egypt; ^4^ Institute of Molecular and Translational Medicine, Faculty of Medicine and Dentistry, and Czech Advanced Technology and Research Institute, Palack University, Olomouc, Czechia; ^5^ Department of Organic Chemistry, University of Valencia, Valencia, Spain; ^6^ Department of Organic and Medicinal Chemistry, Faculty of Pharmacy, University of Sadat City, Menoufiya, Egypt

**Keywords:** spirooxindole, benzimidazole, MEDT, p53, MDM2 inhibitors, NF-κB, CDK (cyclin-dependent kinase)

## Abstract

This study discusses the synthesis and use of a new library of spirooxindole-benzimidazole compounds as inhibitors of the signal transducer and activator of p53, a protein involved in regulating cell growth and cancer prevention. The text includes the scientific details of the [3 + 2] cycloaddition (32CA) reaction between azomethine ylide **7a** and ethylene **3a** within the framework of Molecular Electron Density Theory. The mechanism of the 32CA reaction proceeds through a *two-stage one-step* process, with emphasis on the highly asynchronous transition state structure. The anti-cancer properties of the synthesized compounds, particularly **6a** and **6d**, were evaluated. The inhibitory effects of these compounds on the growth of tumor cells (MDA-MB 231 and PC-3) were quantified using IC_50_ values. This study highlights activation of the p53 pathway by compounds **6a** and **6d**, leading to upregulation of p53 expression and downregulation of cyclin D and NF-κB in treated cells. Additionally, we explored the binding affinity of spirooxindole analogs, particularly compound **6d**, to MDM2, a protein involved in regulation of p53. The binding mode and position of compound **6d** were compared with those of a co-crystallized standard ligand, suggesting its potential as a lead compound for further preclinical research.

## Graphical Abstract

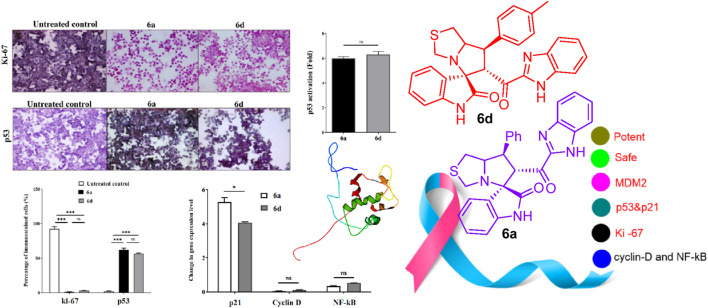

## 1 Introduction

Many diseases have become more common as a result of natural, industrial, and economic issues. Cancer is the second-leading cause of death worldwide, affecting health in all societies. Unfortunately, it is a tissue-level disease, which presents a major challenge in specific diagnosis and treatment efficacy. Prostate, lung, and colorectal cancers are the most common cancers in men worldwide, accounting for 46% of all newly diagnosed cancers in 2021. Breast cancer is the most common cancer in women worldwide, representing 30% of new cases diagnosed in 2021 (Siegel et al., 2021). Cancer is caused by successive mutations in genes that alter cellular functions proliferation and apoptosis (Chaudhry et al., 2022).

p53 is one of the most-studied tumor-suppressor proteins (Kandoth et al., 2013). Mutations in this protein are found in 60% of human cancers. Changing the DNA binding domain inhibits p53 activity as a transcription factor (Janjua, 2004; Estrada-Ortiz et al., 2016); there is evidence to suggest that restoration or reactivation of p53 function has significant therapeutic benefits (Di Agostino et al., 2006; Demma et al., 2010). The p53 pathway is inactivated in the remaining tumors either by downregulation of p53 cooperators such as ARF or upregulation of p53 inhibitors such as mouse double-minute proteins (MDM2 and MDMX) or downregulation of p53 cooperators such as ARF (Hsu and Ip, 2011). Mutations in p53 and upregulation of MDM2 do not typically occur in the same tumor, indicating that MDM2 overexpression is an effective pathway for inactivating p53 function in tumorigenesis. MDM2 inhibits the N-terminal transactivation domain (TAD) of p53 and promotes p53 degradation via the ubiquitin–proteasome system (E3 ligase activity). Substantial data have confirmed that MDM2 is the central node of the p53 pathway. Genetic and biochemical researchers have mapped MDM2-p53 interaction sites to the 106–amino acid-long N terminal domain of MDM2 and the N-terminus of the transactivation domain of p53. Interaction between p53 and MDM2 involves four key hydrophobic residues (Phe 19, Leu 22, Trp 23, Leu 26) in a short amphipathic helix formed by p53 and a small but deep hydrophobic pocket in MDM2. Atomic-level understanding of the MDM2–p53 interaction through x-ray crystallography provides a solid foundation for structure-based design of nonpeptidic, small-molecule antagonists of this interaction (Chen et al., 1993; Kussie et al., 1996). Herein, we set our design rationale to tailor new MDM2 inhibitors endowed with nuclear factor (NF)-κB inhibitory potential to maximize p53 induction capacity (such as enhancing the expression of p21-arrested cell cycle). Previous studies have illustrated that p53 acts as a unique regulator for suppressing NF-κB and aids in preventing its binding to promotor DNA binding sites (Murphy et al., 2011) NF-κB may become overactive in cancer cells because p53 activity is lost (Murphy et al., 2011). NF-κB is an essential transcription factor for expressing several key genes for tumor progression, angiogenesis, and metastasis (Xia et al., 2014; Pires et al., 2017) Thus, it is critical to evaluate the impact of these spirooxindoles on both key transcription factors (p53 and NF-κB).

**FIGURE 1:**
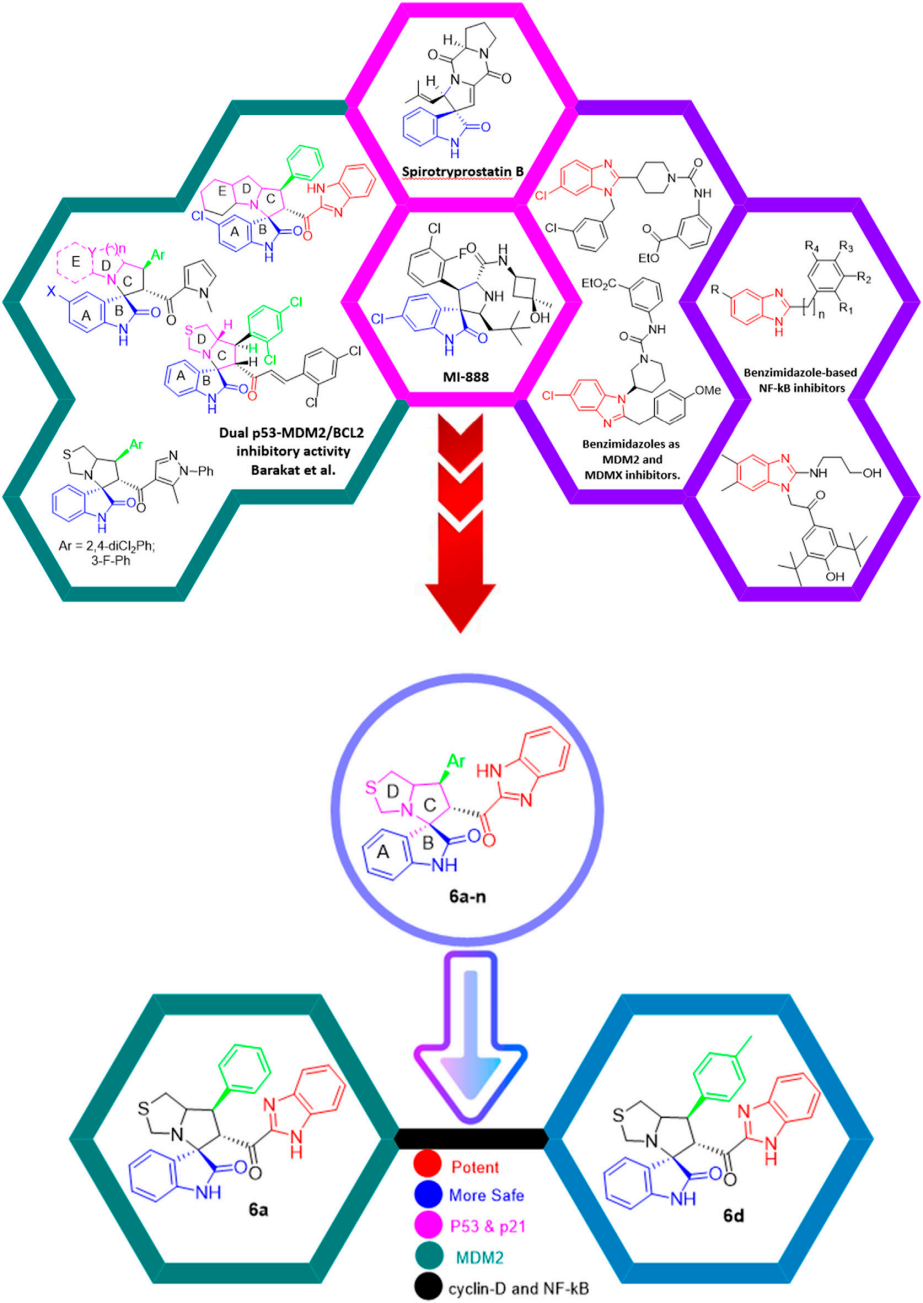
Rationale design of scaffold **6a-n** from naturally occurring or synthetic spirooxindole, benzimidazoles and our previous works.

Several examples based on spirooxindoles (Islam et al., 2023) and benzimidazole core structures have been identified for binding with the MDM2 receptor and activating p53 (Figure 1) (Vassilev, 2007; Li et al., 2008; Riedinger and Mcdonnell, 2009; Millard et al., 2011; Tovar et al., 2013; Zhao et al., 2013; Zhang et al., 2014; Gollner et al., 2016b; Barakat et al., 2019; Islam et al., 2019; Beloglazkina et al., 2020; Aziz et al., 2021; Lotfy et al., 2021; Barakat et al., 2022). Barakat *et al.* designed a new lead compound based on the core structure of spirooxindole clubbed with a benzimidazole moiety that has been shown to be highly effective against cancer cells targeted as inhibitors of protein–protein interaction between MDM2-p53 genes. This compound also exhibited a potential anti-metastatic effect (Islam et al., 2019; Aziz et al., 2021; Lotfy et al., 2021; Barakat et al., 2022). With this finding and the high potentiality of the benzimidazole nucleus toward cancer treatment-targeted NF-κB (Okolotowicz et al., 2010; Poltz and Naumann, 2012; Boggu et al., 2016; Jonak et al., 2016; Boggu et al., 2017; Dunphy et al., 2018; Błaszczak-Świątkiewicz, 2019), we were encouraged to increase the libraries of these analogs and assess them against different cancer cells. Based on the abovementioned data, the rational design of this work was conceptualized by merging some key features from naturally occurring spirooxoindoles with anticancer activities; reported Benzimidazole-based NF-κB inhibitors; our previous works with dual p53-MDM2/BCL2 (A, B,C,D,E system); and previous spiroxoxindoles–imidazole hybrid with A, B,C,D,E system. The designed compounds contain spirooxoindoles-imidazole with A, B,C,D system.

## 2 Results and discussion

### 2.1 Chemistry

**SCHEME 1:**
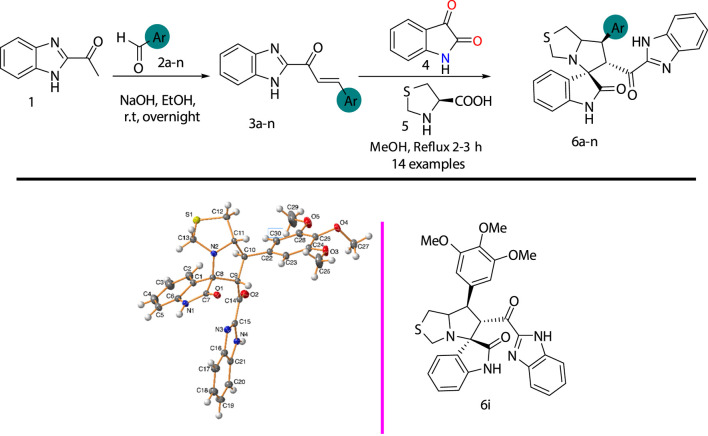
Synthesis of the desired spirooxindole derivative **6a-n;** ORTEP for spirooxindole analog **6i**.

**SCHEME 2:**
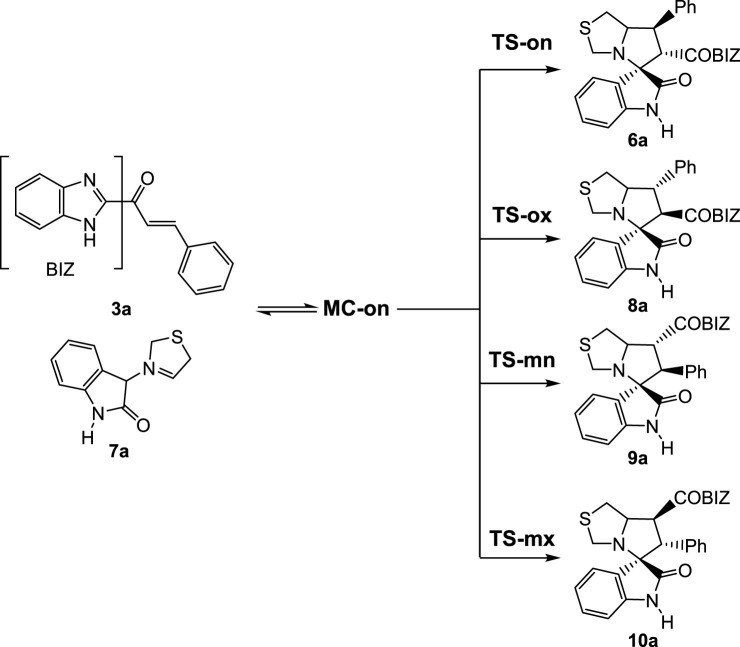
32CA reaction of AY **7a** with the ethylene **3a**.

To increase the library of spirooxindole scaffolds as lead compounds for development of new drugs for cancer treatment targeting MDM2 inhibitors (Alshahrani et al., 2023), new spirooxindole derivatives (**6a–n**) were synthesized by the reaction of chalcones **3a–n** (Supplementary Table S1) with isatin **4** and thiazolidine-4-carboxylic acid **5** in methanol under reflux for 2–3 h, as shown in Scheme 1. The new spiro-derivatives **6a–n** was fully characterized by FT-IR, ^1^H- and ^13^C- NMR, and elemental analysis; spirooxindole analog **6i** was characterized by single-crystal x-ray diffraction analysis (data provided in SI, Supplementary Tables S1–S4). The stereochemistry and absolute configuration were assigned using experimental or theoretical approaches, and confirmed that the [3 + 2] cycloaddition (32CA) reaction proceeded *via* the *ortho/endo* pathway reaction mechanism (Scheme 2).

### 2.2 MEDT study of 32CA reaction between AY 7a and ethylene 3a

The 32CA reaction of AY **7a** with ethylene **3a** yielding *ortho*/*endo* spirooxindole **6a** was theoretically studied within the Molecular Electron Density Theory (MEDT) (Domingo, 2016) to understand its behaviors (Scheme 2).

#### 2.2.1 Study of 32CA reaction of AY **7a** with ethylene **3a**

Owing to the non-symmetry of both reagents, two pairs of *endo* and *exo* stereoisomeric and two pairs of *ortho* and *meta* regioisomeric reaction paths were studied (Scheme 2). Analysis of the stationary points found along the four reaction paths indicated that the 32CA reaction occurs through a one-step mechanism. *ω*B97X-D/6-311G(d,p) relative enthalpies and Gibbs free energies are presented in Table 1. The thermodynamic data are presented in Supplementary Table S7.

**TABLE 1:** *ω*B97X-D/6–311G(d,p) relative enthalpies (ΔH in kcal·mol^-1^), entropies (ΔS in cal·mol^-1^K^−1^), and Gibbs free energies (ΔG in kcal·mol^-1^), with respect to the separated reagents, computed at 337.85 K and 1 atm in methanol, for the stationary points involved in the 32CA reaction of AY **7a** with ethylene **3a**.

	ΔH	ΔS	ΔG
**MC-on**	−18.6	−42.0	−4.4
**MC-mn**	−15.2	−37.9	−2.3
**TS-on**	−11.7	−52.9	6.1
**TS-ox**	−6.1	−50.1	10.9
**TS-mn**	−7.2	−48.6	9.2
**TS-mx**	−4.4	−46.5	11.4
**6a**	−47.9	−48.6	−31.5
**8a**	−44.2	−47.3	−28.3
**9a**	−45.1	−48.1	−28.8
**10a**	−48.3	−48.5	−31.9

A series of molecular complexes (MCs) were also found, in which the two reagents were joined by weak intermolecular interactions. Only the most stable **MC-on** was selected as an energy reference. The distance between the two frameworks at this MC was *ca.* 3.2 Å; **MC-on** was 18.6 kcal·mol^-1^ below the separated reagents (Table 1). Some conclusions can be drawn from the relative enthalpies in methanol given in Table 1: i) the most favorable **TS-on** was 11.7 kcal·mol^-1^ below the separated reagents; if formation of **MC-on** is considered, the activation enthalpy becomes positive by 6.9 kcal·mol^-1^; ii) this 32CA reaction is completely *endo* stereoselective as **TS-ox** was 5.7 kcal·mol^-1^ above **TS-on**; iii) this 32CA reaction is completely *ortho* regioselective as **TS-mn** was 4.5 kcal·mol^-1^ above **TS-on**. *Endo* stereoselectivity and *ortho* regioselectivity are in complete agreement with the experimental outcomes; iv) this 32CA reaction was strongly exothermic as spirooxindole **6a** was 47.9 kcal·mol^-1^ below the separated reagents. Consequently, spirooxindole **6a** was formed via kinetic control.

**FIGURE 2:**
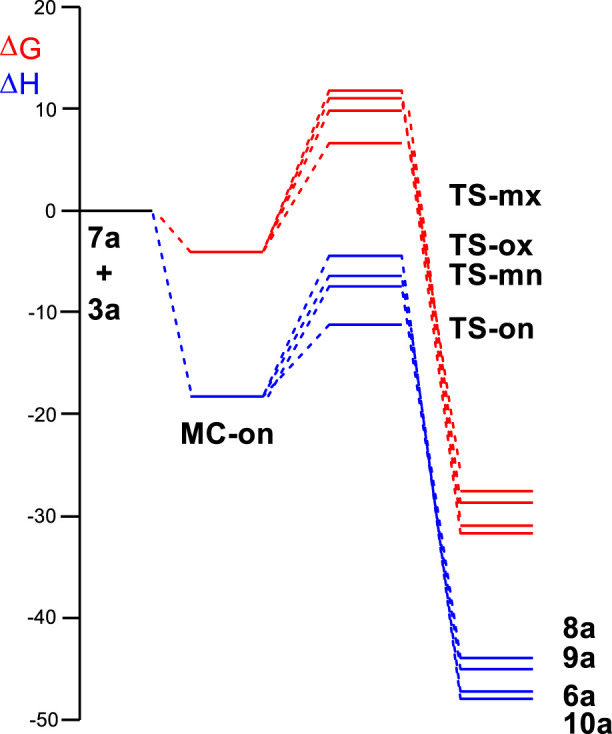
ωB97X-D/6–311G(d,p) enthalpy, in blue, ΔH in kcal mol^–1^, and Gibbs free energy, in red, ΔG in kcal mol^–1^, profiles, in methanol at 65°C, for the 32CA reaction of AY **7a** with the ethylene **3a**.

The enthalpy and Gibbs free energy profiles associated with the four competitive reaction paths are shown in Figure 2. Inclusion of the thermal corrections and entropies to enthalpies increases the relative Gibbs free energies by 14.2–17.9 kcal·mol^-1^ as a consequence of the unfavorable relative entropies associated with this bimolecular process, which were between −42.0 cal·mol^-1^·K^−1^ and −52.9 cal·mol^-1^·K^−1^. Formation of **MC-on** was exergonic by 4.4 kcal·mol^-1^. The activation Gibbs free energy associated with the 32CA reaction of AY **7a** with ethylene **3a** *via* **TS-on** increased to 10.5 kcal·mol^-1^; formation of spirooxindole **6a** was exergonic by 31.5 kcal·mol^-1^. Considering the activation Gibbs free energies, this 32CA reaction is completely *endo* stereoselective and *ortho* regioselective as **TS-ox** and **TS-mn** are 4.7 kcal·mol^-1^ and 3.1 kcal·mol^-1^, respectively, above **TS-on** (Figure 2).

**FIGURE 3:**
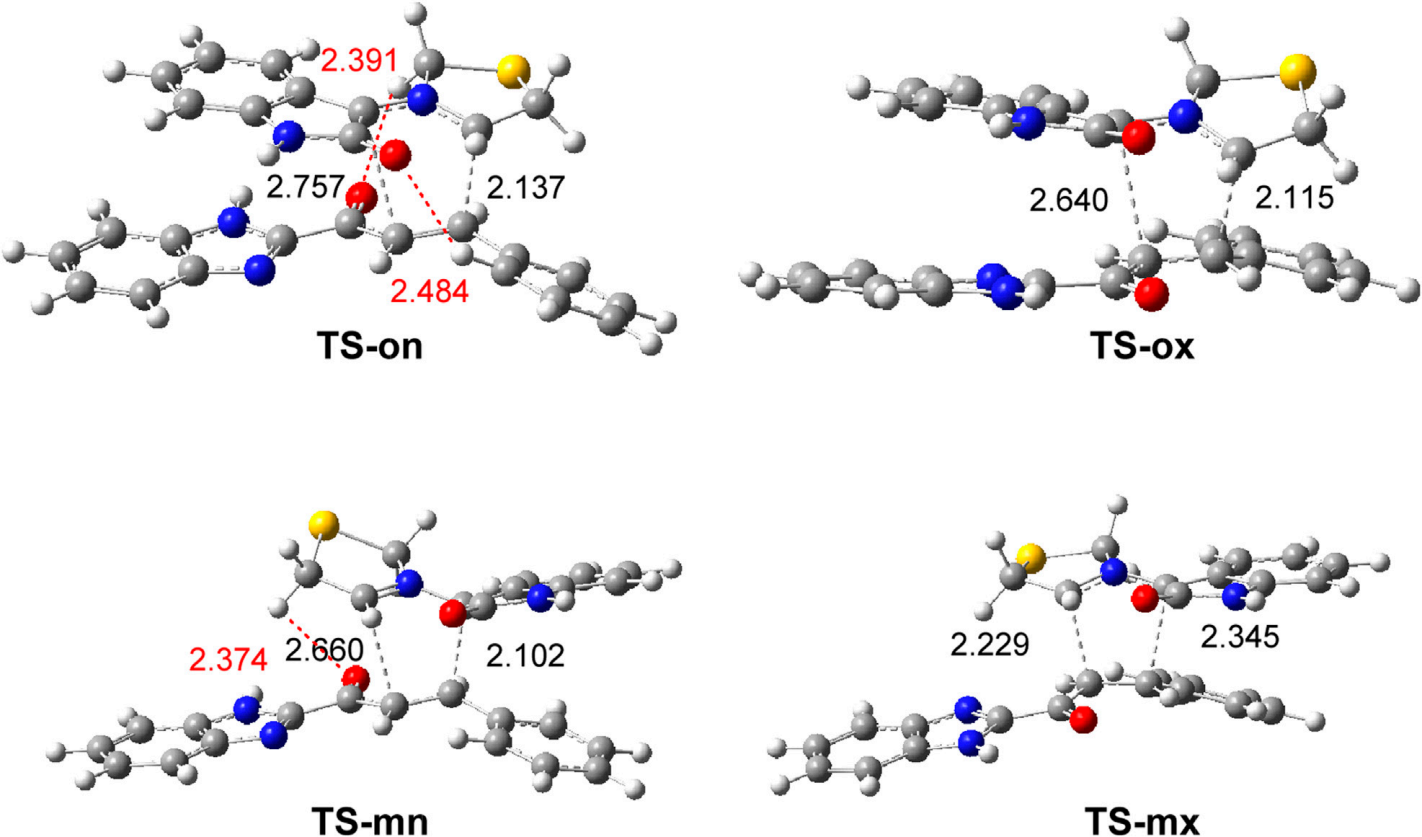
*ω*B97X-D/6–311G(d,p) geometry of the TSs involved in the 32CA reaction of AY **7a** with ethylene **3a**, optimized in methanol. The distances are given in Angstrom. HB distances are given in red.

The geometries of the four TSs optimized in methanol are shown in Figure 3. The C−C distances between the four interacting carbons in the four TSs indicate that aside from the most unfavorable **TS-mx**, the other three TSs correspond to asynchronous C−C single-bond formation processes in which the shorter C−C distance corresponds to participation of the most electrophilic β-conjugated C4 carbon of ethylene **3a**. At the most favorable **TS-on**, the C−C distances between the two pairs of interacting carbons, 2.137 Å (C3−C4) and 2.757 Å (C1−C5), indicate that this TS is associated with a high asynchronous C−C single-bond formation process. Analysis of the intrinsic reaction coordinates (IRC) (Fukui, 1970) associated with the highly asynchronous **TS-on** indicates that this 32CA reaction occurs through a non-concerted *two-stage one-step* mechanism (Domingo et al., 2008), in which the formation of the second C−C single bond begins after the first C−C single bond is completely formed.

Detailed analysis of the geometry of **TS-on** shows that one of the hydrogens of the benzene framework of ethylene **3a** is located at 2.484 Å of the carbonyl oxygen of AY **7a**, and one hydrogen of the dihydrothiopyrrole ring of AY **7a** is located at 2.391 Å of the carbonyl O7 oxygen of ethylene **3a** (Figure 3). These distances suggest the presence of two hydrogen bonds (HBs) between the hydrogen and oxygen centers. However, analysis of the geometry of the regioisomeric **TS-mn** shows the presence of only one HB between carbonyl O7 oxygen of ethylene **3a** and dihydrothiopyrrole hydrogen of AY **7a**, with H−O distances of 2.374 Å (Figure 3).

Global electron density transfer (GEDT) analysis (Domingo, 2014) at the most favorable **TS-on** was used to assess the polar character of this 32CA reaction. GEDT values less than 0.05 e correspond to non-polar processes; values greater than 0.20 e correspond to polar processes. The high GEDT value found at **TS-on** (0.23 e), a consequence of the supernucleophilic character of AY **7a** (*N* = 4.39 eV) and the strong nucleophilic character of ethylene **3a** (ω **=** 2.39 eV) (see Conceptual DFT (CDFT) analysis (Parr, 1983; Domingo et al., 2016) of the reagents in the Supplementary Material), indicates that this 32CA reaction is highly polar. The flux of the electron density, from AY **7a** to ethylene **3a**, classifies this 32CA reaction as a forward electron density flux (FEDF) (Domingo et al., 2020), in agreement with the analysis of the CDFT indices.

#### 2.2.2 What is the origin of the *ortho* regioselectivity?

**FIGURE 4:**
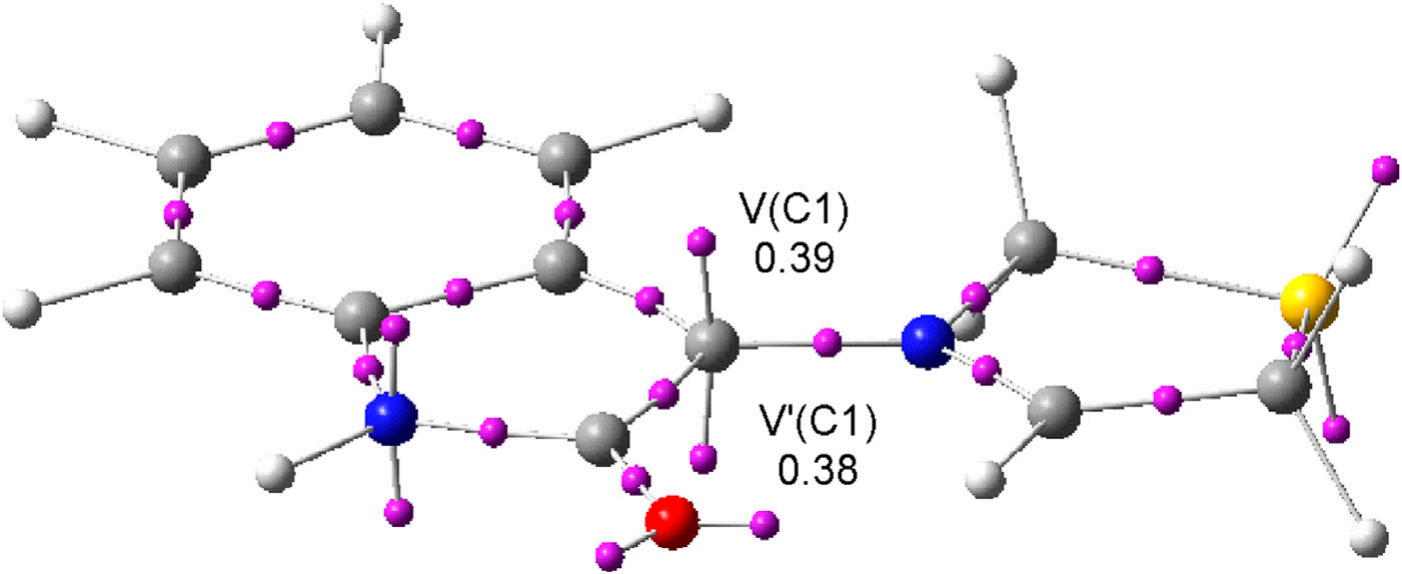
*ω*B97X-D/6–311G(d,p) ELF basin attractor positions of AY **3a**. The populations of the V(C1) and V′ (C1) monosynaptic basins are given in average number of electrons, e.

Topological analysis of the electron localization function (ELF) (Becke and Edgecombe, 1990) of AY **7a** showed the presence of two monosynaptic basins, V(C1) and V’ (C1), integrating a total of 0.76 e at the C1 carbon, characterizing this three-atom-component as a *pseudo*(*mono*)*radical* species (Ríos-Gutiérrez et al., 2022) (Figure 4). Analysis of the nucleophilic P_k_^−^ Parr functions (Domingo et al., 2013) of AY **7a** indicates that the C1 carbon is more nucleophilic than the C3 carbon (see CDFT analysis of the reagents in the Supplementary Material). Consequently, the 32CA reaction of AY **7a** with ethylene **3a** is expected to be *meta* regioselective (Ríos-Gutiérrez et al., 2022).

A recent MEDT study on the unexpected *ortho* regioselectivity in the 32CA reactions of AYs derived from L-proline showed that the presence of two HBs at the **MC-on**, which are enhanced at the corresponding **TS-on**, can be responsible for the *ortho* regioselectivity of these 32CA reactions (Domingo et al., 2022).

**FIGURE 5:**
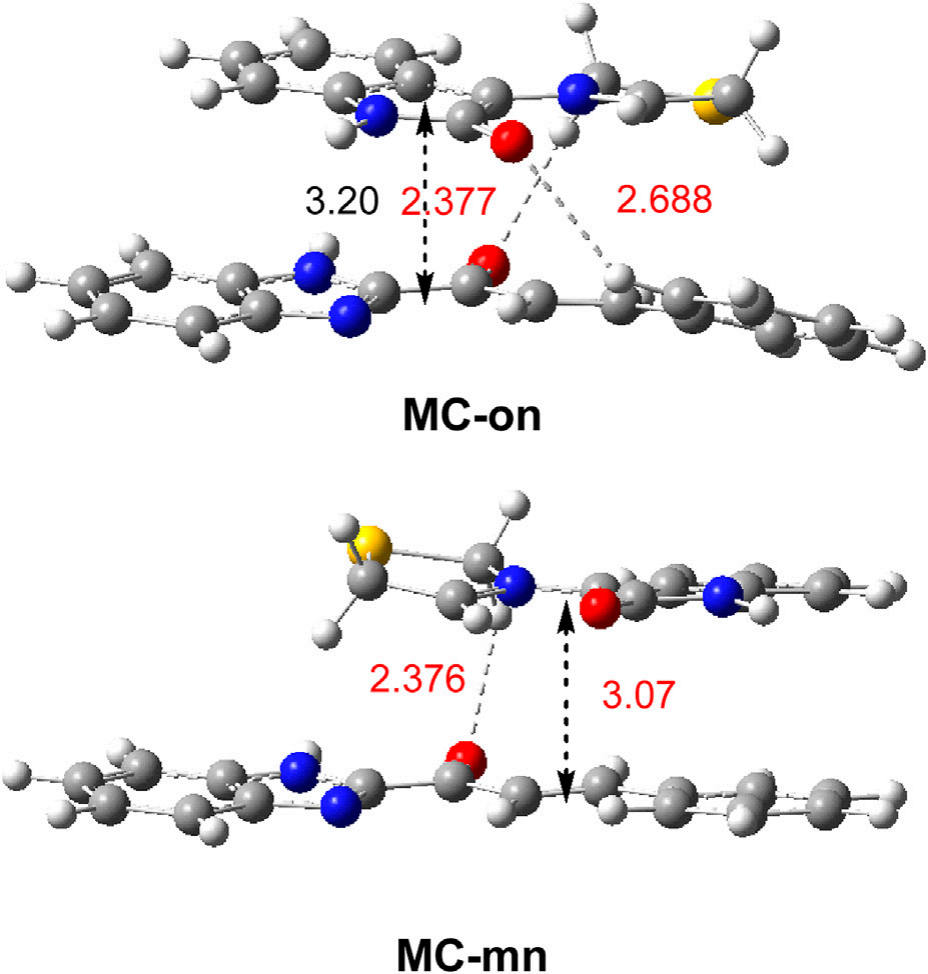
*ω*B97X-D/6–311G (d,p) optimized geometries of **MC-on** and **MC-mn**. Distances in methanol are given in angstroms, Å. HB distances are given in red.

**MC-on** was 3.4 kcal mol^-1^ more stable than **MC-mn** (Table 1). Analysis of the geometries of the two MCs shows the presence of an HB between one hydrogen of the CH_2_ methylene continuous with the sulfur of AY **3a** and the carbonyl O7 oxygen of ethylene **7a**, with an O−H distance of 2.37 Å (Figure 5). **MC-on** shows an additional HB between a hydrogen of the aromatic ring of ethylene **3a** and the oxygen atom of AY **7a,** with an O−H distance of 2.69 Å (Figure 5). Formation of this HB required twisting of the benzene ring by 15.9°.

These two HBs, which are also present in the most stable **TS-on** (Figure 3), may account for the *ortho* and *endo* selectivity found in this 32CA reaction. The enthalpy difference between **MC-on** and **MC-mn (**3.4 kcal mol^-1^) was increased to 4.5 kcal·mol^-1^ at the TSs as a consequence of stronger HB interactions.

An atom-in-molecules (AIM) (Richard et al., 1982) topological analysis of the electronic structure of the most favorable **TS-on** characterizes the two HBs responsive to the *ortho* reactions (see AIM topological analysis of the electronic structure of the most favorable **TS-on** in the Supplementary Material Table S6).

### 2.3 Biological evaluation and MTT assay

Initially, the target compounds of the spirooxindole analogs **6a–n** were submitted to NCI for screening against 60 panels of cancer cells. Preliminary results showed promising feedback for the synthesized compounds of spirooxindole analogs (SI, Supplementary Table S8). For example, compounds **6a–n** perfectly inhibited the growth of leukemia cells (CCRF-CEM; HL-60 (TB); K-562; MOLT-4; RPMI-8226, SR), with a GI% exceeding 80% except for compounds **6b**, **6g**, and **6l**, which showed a smaller GI% (75%) for MOLT-4, RPMI-8226, and SR cancer cells. In non-small-cell lung cancer cells (A549/ATCC, EKVX, HOP-92, HOP62, NCI-H226, NCI-H23, NCI-H322M, NCI-H460, NCI-H460), the synthesized compounds showed complete growth inhibition, with the exception of **6g** and **6l**, which were the most reactive. We also observed that compounds **6g** and **6l** exhibited growth inhibition of less than 80% in CNS cancer cells (SF-268, SF-295, SF-539, SNB-19, SNB-75, U251). The GI% of melanoma cells was completely inhibited by these compounds. For ovarian cancer cells (IGROVI), compounds **6b, 6c, 6d, 6g**, and **6l** showed less reactivity, with a GI% from 55% to 75%. The other compounds showed excellent growth inhibition. For renal cancer cells (UO-31), the compounds exhibited less reactivity than with other renal cancer cells (786–0, A49, ACHN, CAKI-1, RXF393, SN12C, TK-10, DU-145). For breast cancer cells, complete growth inhibition was observed; however, compounds **6b–d, 6g, 6h**, and **6m** were less reactive against T-47D cancer cells, with a GI% between 54% and 80%.

**FIGURE 6:**
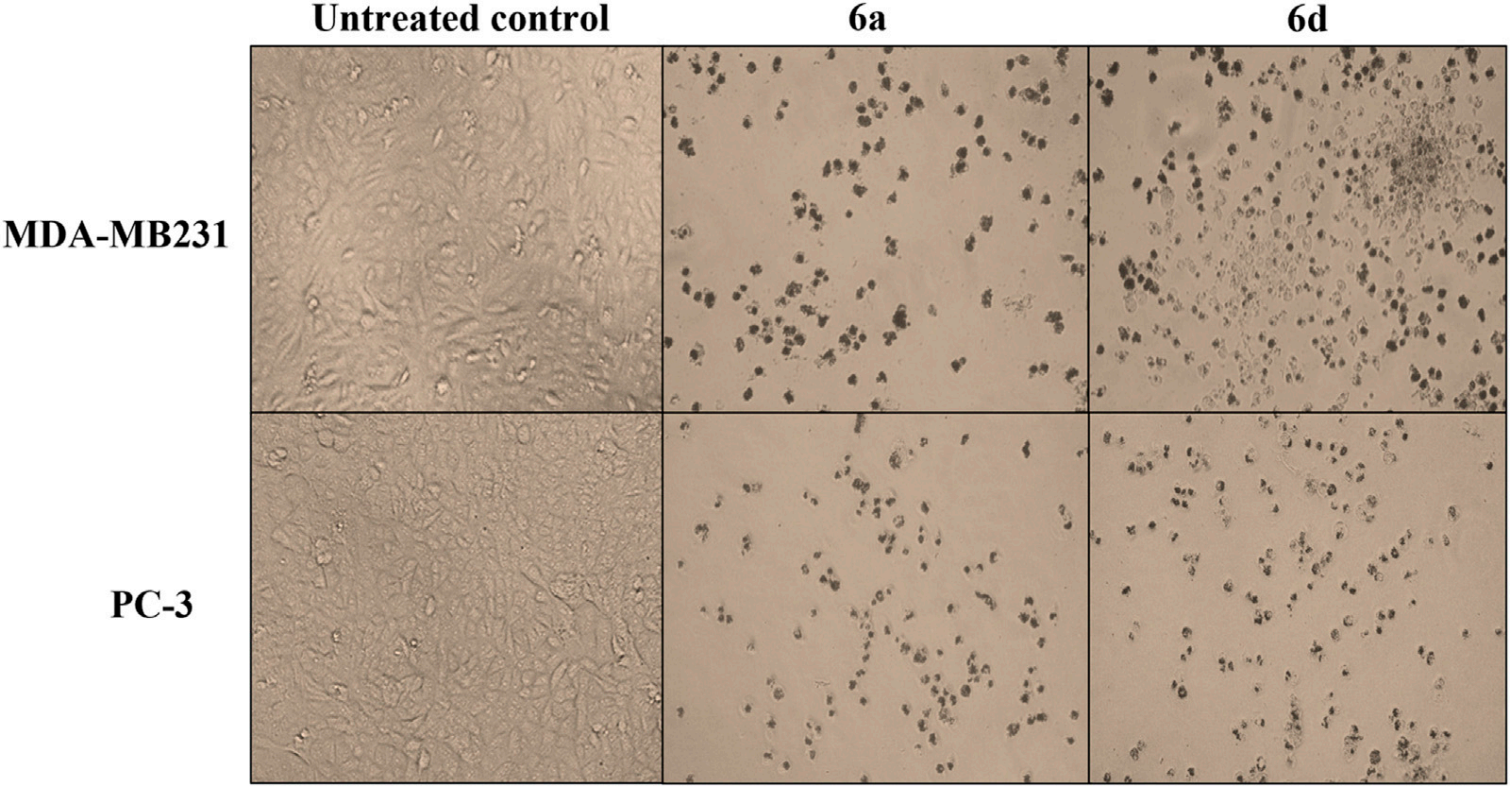
Morphological alteration of the most active anticancer compounds-treated MDA-MB 231 and PC-3 cells in comparison with the untreated control cells.

The percentage of wi-38 viability and the growth inhibition percentages of MDA-MB231 and PC-3 cells after incubation with 5 µM of different tested compounds were assessed using the MTT assay (Supplementary Table S9). From Supplementary Table S9, the viability of normal human lung cells (wi-38) exceeded 81% after incubation with 5 µM of **6a** and **6d** compared to all other tested compounds, producing wi-38 cell death over 50%. The safest compound was **6a,** which did not affect the cell viability of wi-38 (_~_96%). Compounds **6a** and **6d** produced maximum viability on wi-38 and triggered death (>50%) in human cancer cells (MDA-MB 231 and PC-3) (Supplementary Table S9). The values of the IC_50_ (µM) of **6a-n** on Wi-38 viability and the growth of MDA-MB 231, and PC-3 cells are summarized in Table 2. This powerful anti-cancer effect was supported by severe morphological alterations (cell shrinkage and loss of normal spindle shape) in treated cancer cells compared with untreated cancer cells (Figure 6).

**TABLE 2:** The estimated IC_50_ (*µ*M) of **6a-n** on Wi-38 viability and the growth of MDA-MB 231, and PC-3 cells.

Chemical structure	Wi-38	MDA-MB 231	PC-3
	IC_50_ (μM)		
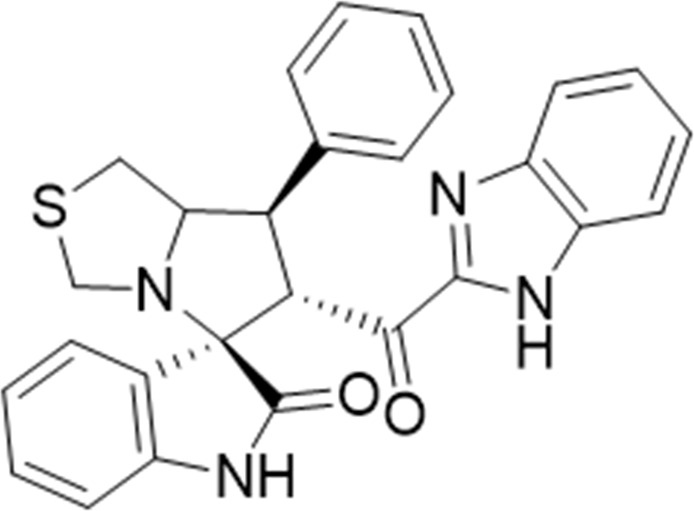 **6a**	22.163 ± 1.586	2.968 ± 0.047	4.107 ± 0.167
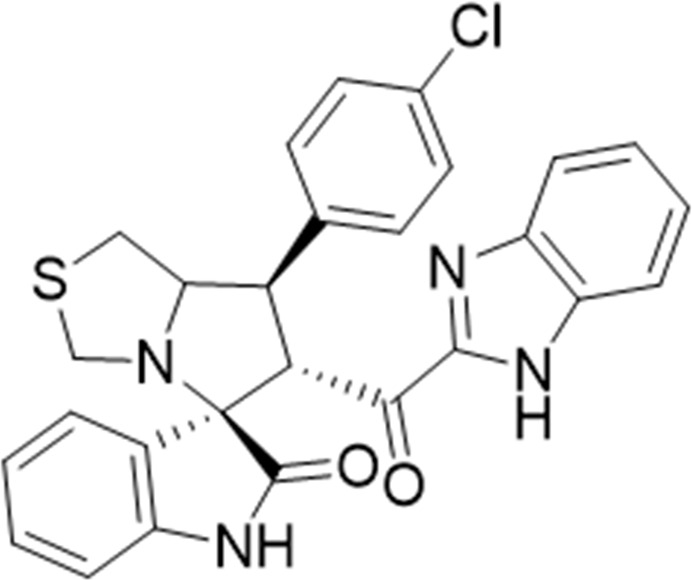 **6b**	4.962 ± 0.117	4.757 ± 0.265	4.819 ± 0.000
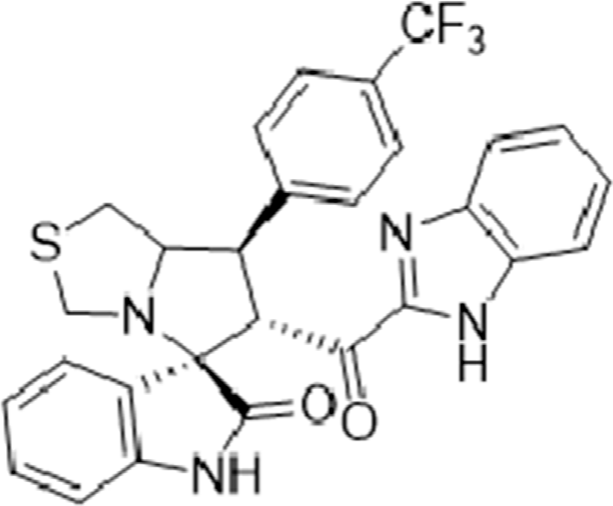 **6c**	12.887 ± 1.512	3.341 ± 0.210	4.151 ± 0.232
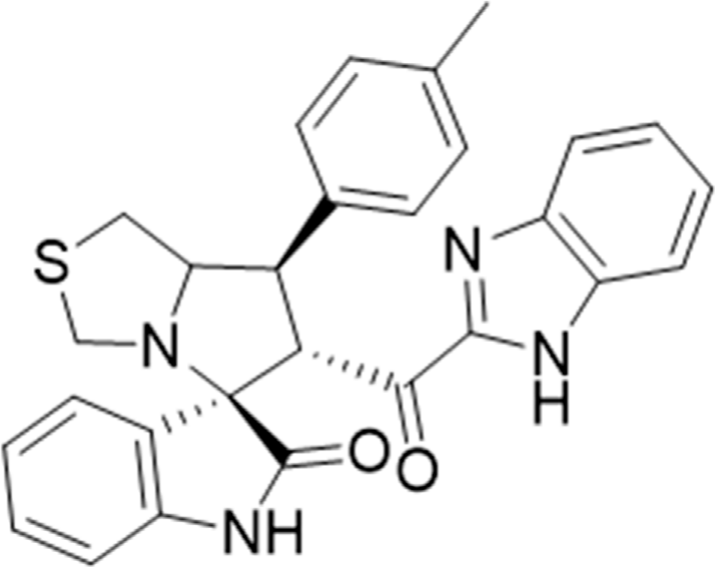 **6d**	5.180 ± 0.138	4.303 ± 0.137	4.294 ± 0.054
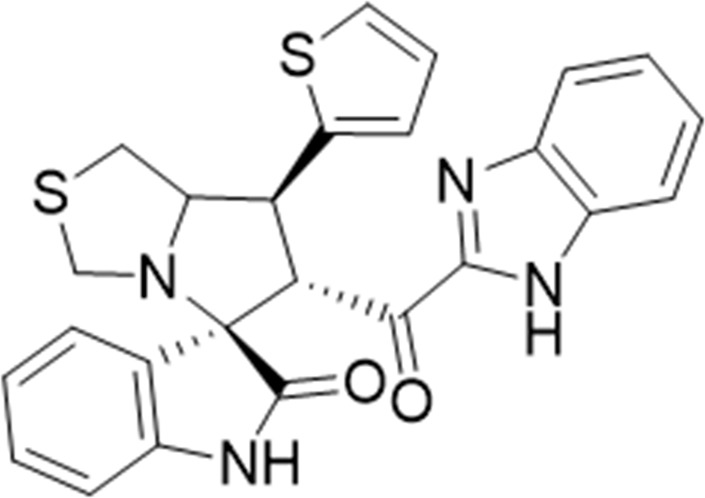 **6e**	4.752 ± 0.008	5.605±	5.432 ± 0.098
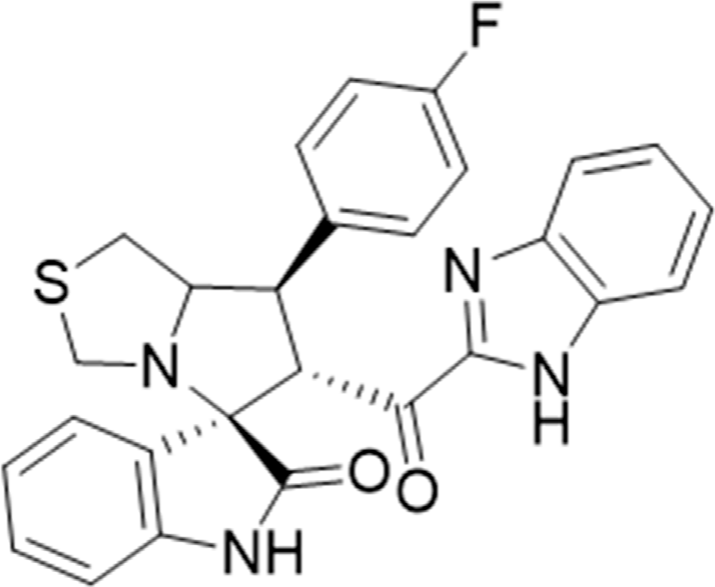 **6f**	4.166 ± 0.076	4.407 ± 0.448	4.593 ± 0.132
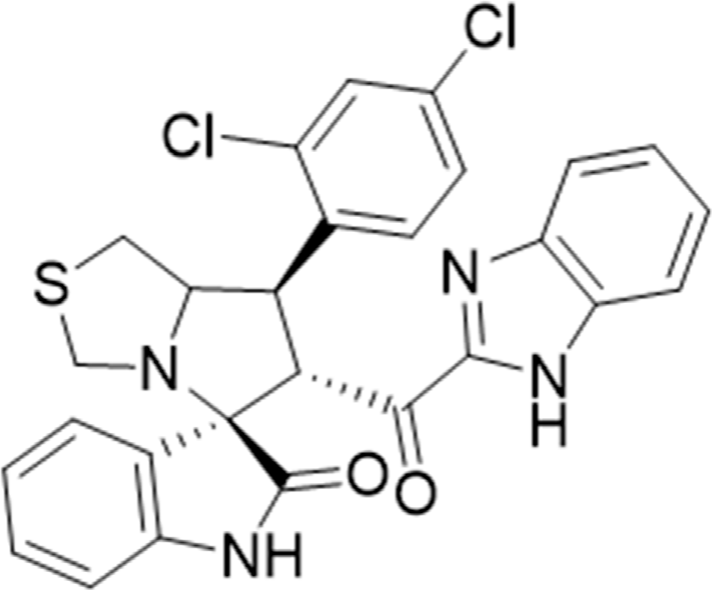 **6g**	5.106 ± 0.191	6.216 ± 0.000	6.191 ± 0.191
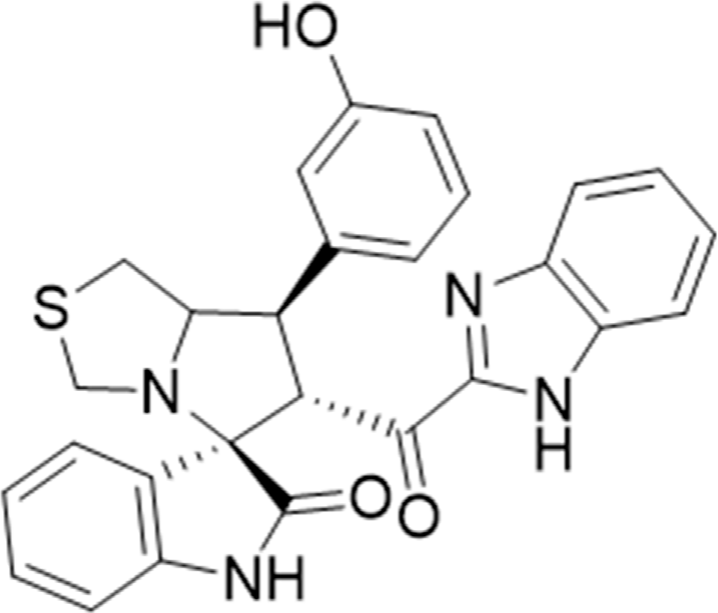 **6h**	4.408 ± 0.07	5.442 ± 0.167	5.041 ± 0.316
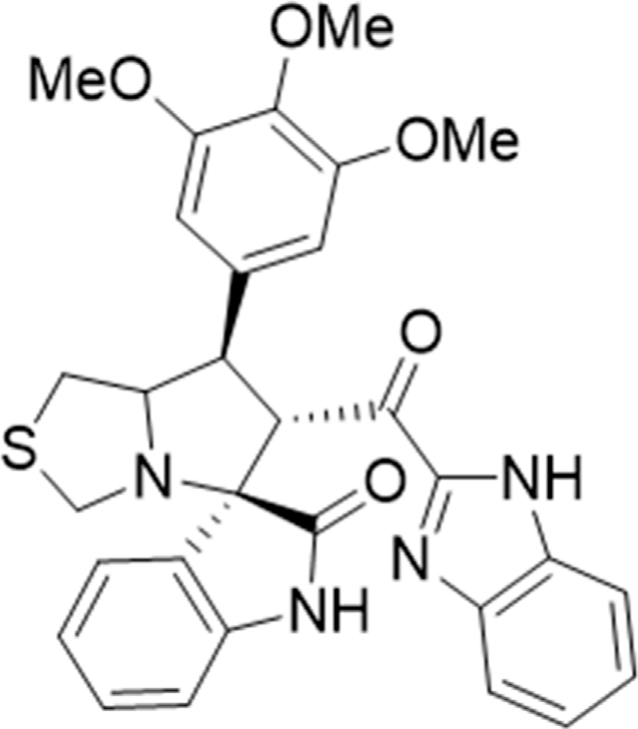 **6i**	4.081 ± 0.201	6.680 ± 0.463	6.017 ± 0.106
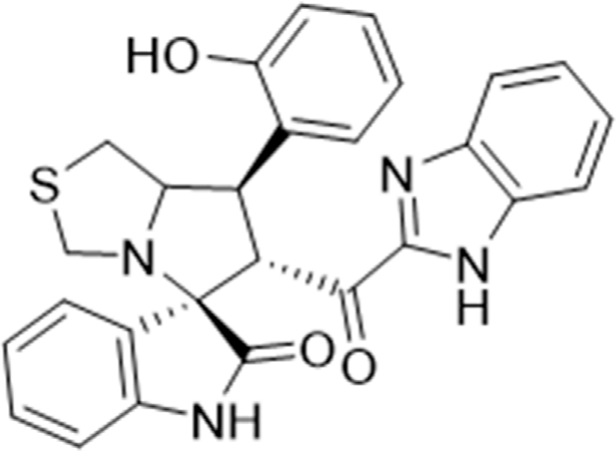 **6j**	3.998 ± 0.129	4.665 ± 0.009	4.543 ± 0.163
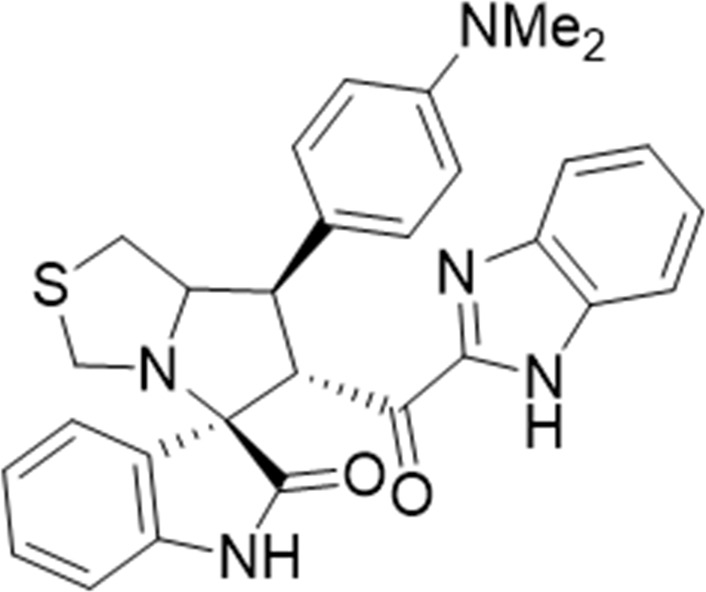 **6k**	3.950 ± 0.319	3.995 ± 0.249	4.160 ± 0.036
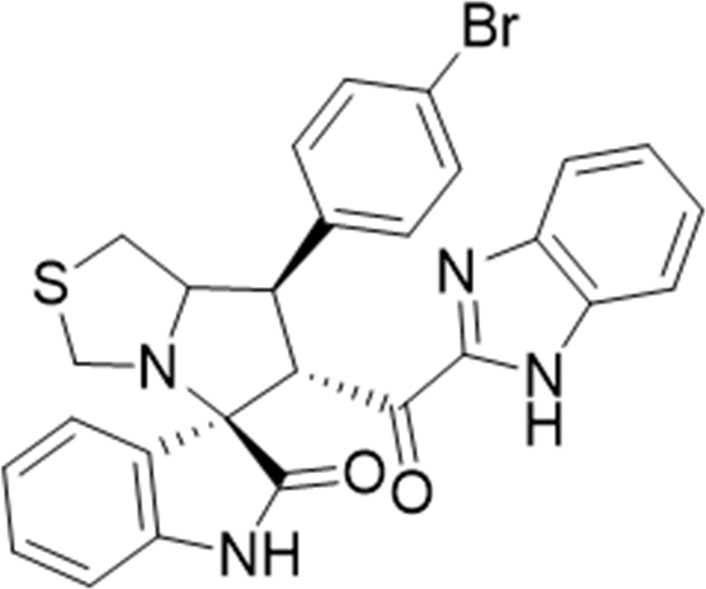 **6l**	6.259 ± 0.443	4.510 ± 0.203	4.627 ± 0.116
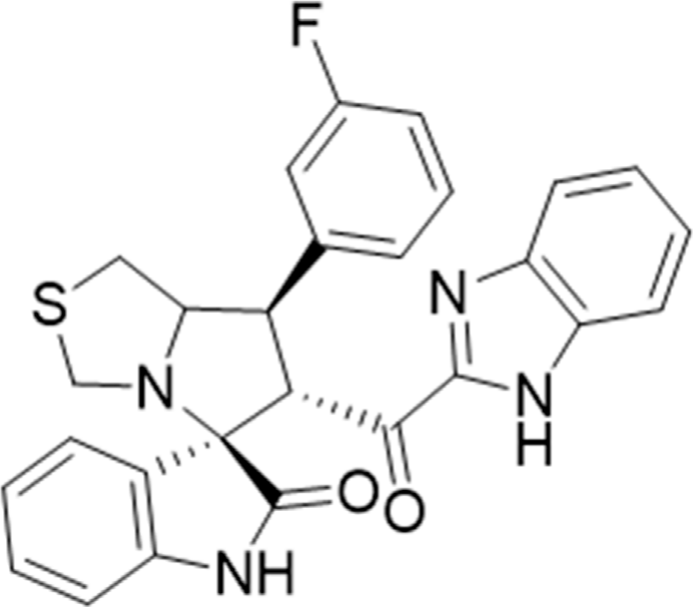 **6m**	4.650 ± 0.127	4.733 ± 0.224	4.766 ± 0.132
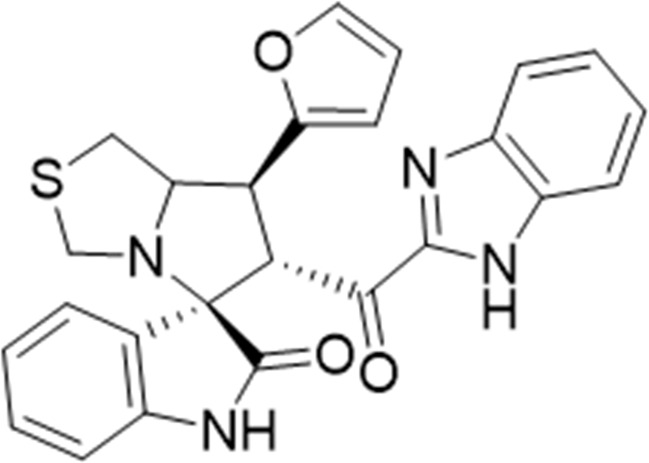 **6n**	4.171 ± 0.166	6.143 ± 0.246	6.247 ± 0.533

**FIGURE 7:**
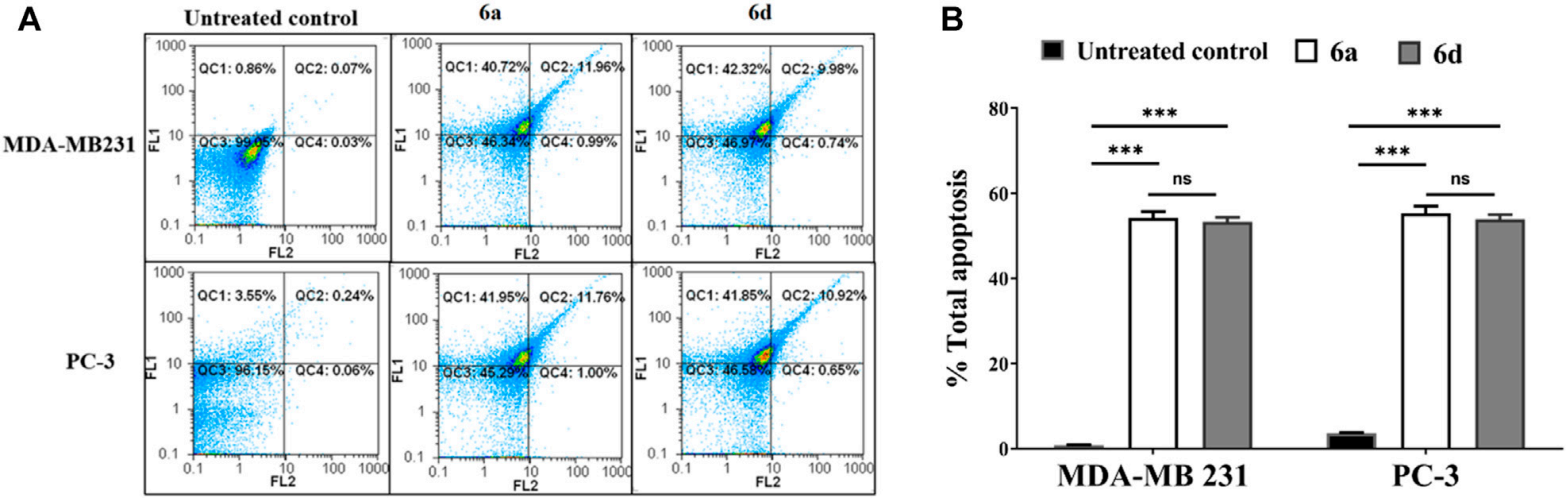
Flow analysis of the most active compounds-treated MDA-MB 231 and PC3 cell lines after dual staining with annexin and propidium iodide. **(A)** Flow charts and **(B)** the total percentage of the apoptotic cell population in the treated cancer cells. Data considered statistically significant at *p* ≤ 0.05*, ≤0.005**, and ≤0.001***. ns: Not statistically significant.

**FIGURE 8:**
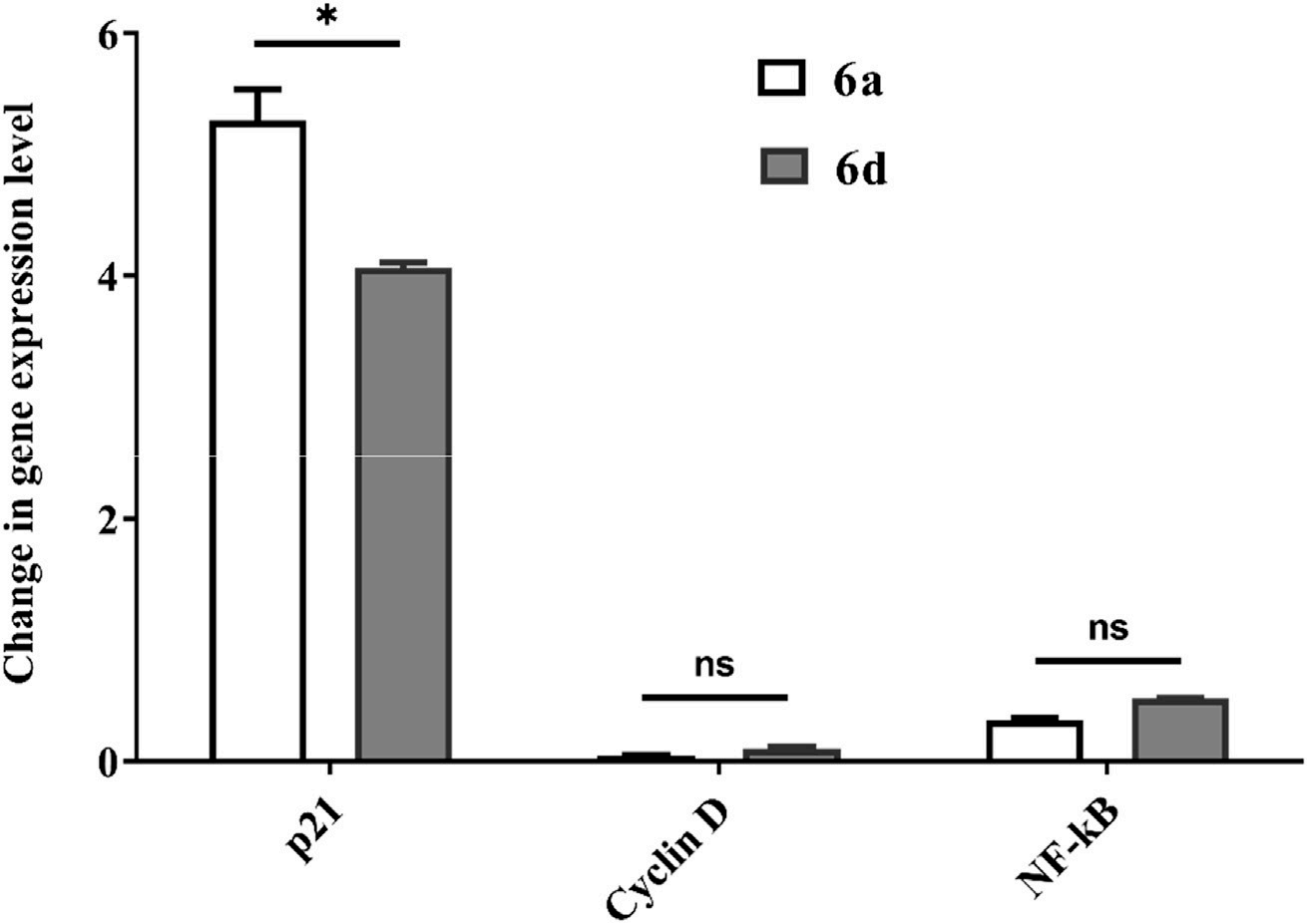
Relative change in gene expression of p21, cyclin-D, and NF-κB in the **6a** and **6d**-treated MDA-MB 231 cells. Data considered statistically significant at *p* ≤ 0.05*, ≤0.005**, and ≤0.001***. ns: Not statistically significant.

Figures 7A, B shows that **6a-** and **6d**-treated cancer cells demonstrated a higher percentage of annexin-stained cells than untreated cells. Furthermore, **6a** and **7d** induced apoptosis-dependent death (53.89%–55.34%) in treated breast- and prostate-cancer cell lines. This effect may be attributed to the potency of **6a** and **6d** for upregulating p21 expression by greater than three times and downregulating the expression level of cyclin D and NF-κB in treated MDA-MB 231 cells compared to untreated cells (Figure 8). **6a** had the most significant effect on induction of p21 expression (5.3 times) and suppression of cyclin D and NF-_k_B gene expression compared with **6d** (*p* < 0.05).

**FIGURE 9:**
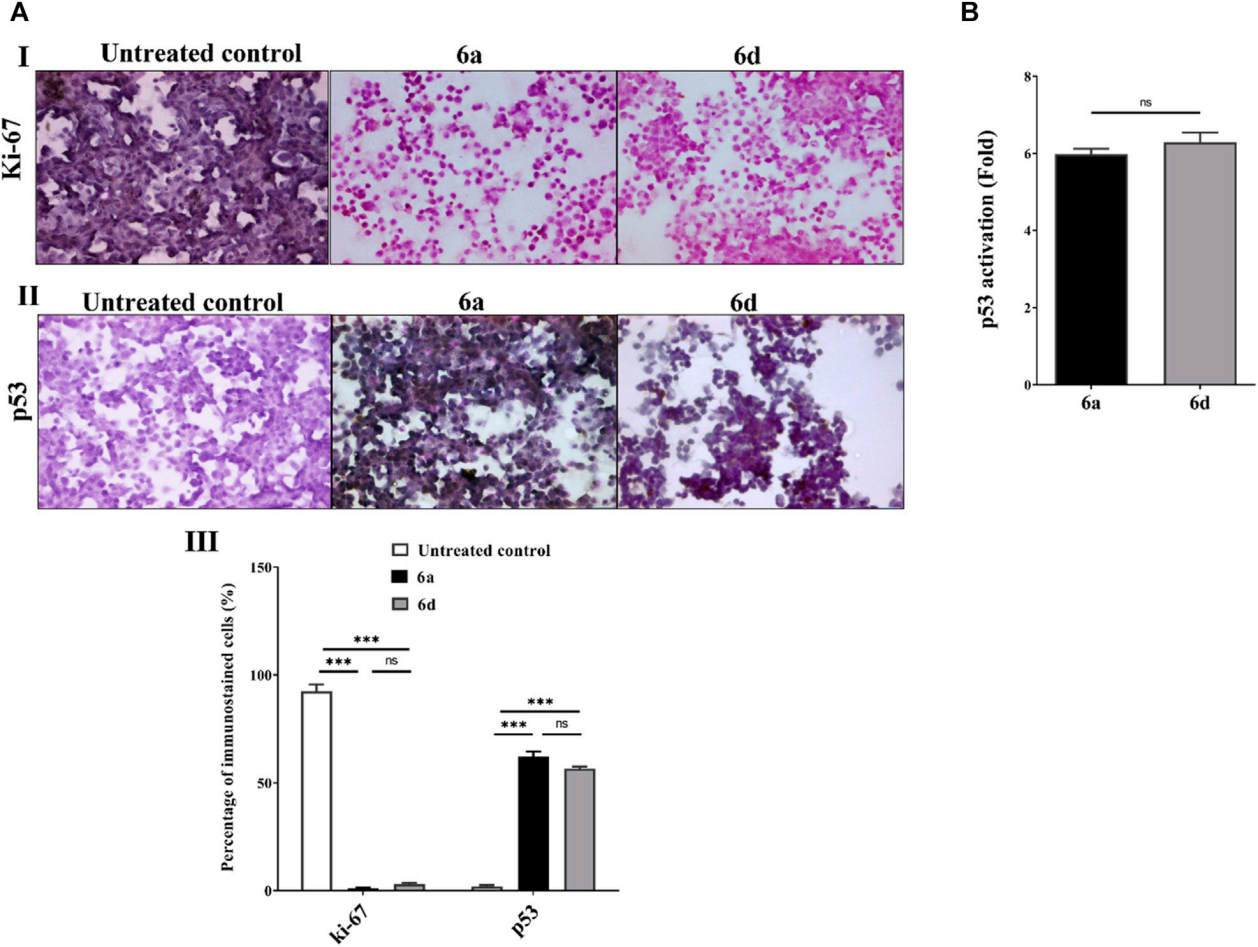
Immunohistochemical staining Ki-67 and p53 as well as fold increment in p53 activation in MDA-MB 231 cells. **(A)** Immunostaining pattern of **(I)** Ki-67 and (**II**) p53 in the untreated and **6a** and **6d**-treated MDA-MB 231 cells (magnification×100) with (**III**) representative percentages of positive immunostaining MDA-MB 231 cells **(B)** Fold activation of p53 transcription factor in **6a** and **6d**-treated MDA-MB 231 cells relative to the untreated cells. Data considered statistically significant at *p* ≤ 0.05*, ≤0.005**, and ≤0.001***. ns: Not statistically significant.

Figure 9A shows the immunohistochemistry results for the proliferation marker (Ki-67) and apoptosis marker (p53) in the MDA-MB 231 cell line after treatment for 72 h with **6a** and **6d**. These compounds reduced Ki-67 levels (from 82.425% to 1.18% and 3.03%, respectively), as indicated by the increase in purple-stained cells in comparison with brown-stained untreated control cells (Figures 9AI, III). Figures 9AII, III show that **6a** and **6d** increased the protein levels of p53 by 60.2% and 54.7%, respectively, compared to the untreated MDA-MB 231 cells, which mostly lacked brown p53-nuclear staining. Moreover, the active p53 transcription factor was assessed in the treated MDA-MB 231 cells compared to untreated cells by quantifying the p53 bound to a target oligonucleotide-coated plate. Both compounds exhibited high efficiency for p53 activation, 5.983 ± 0.142 and 6.287 ± 0.254, respectively (Figure 9B). This may be due to their inhibitory potency against MDM2, which limits its degradation.

Some of the synthesized spirooxindoles based on benzimidazole were selected for further MDM2 binding affinity using the MST assay. The results are summarized in Table 3; the binding curve is presented in the Supplementary Information. Compounds **6a, 6b, 6d, 6e, 6h, 6i**, and **6l** exhibited moderate to excellent binding affinity with the MDM2 receptor, with a range of *K*_*D*_ = 6.68–113 *µ*M (SI; Supplementary Figures S3–S9). The most active compound was **6h** (*K*_*D*_ = 6.68 *µ*M); the spirooxindole-based benzimidazole had a benzene ring with a hydroxyl group in the *meta*-position for further binding interaction with the key amino acid. Having electron-donating groups such as methyl or trimethoxy groups on the benzene ring (**6d** and **6i)** showed good binding, with *K*_*D*_ = 17.4 *µ*M and 21 μM, respectively. However, the presence of halogens such as chlorine or bromine atoms decreased the binding affinity with MDM2 to 38.2 *µ*M and 49.9 *µ*M for compounds **6b** and **6l,** respectively. Without any substitution on the benzene ring (compound **6a**) or change to a heterocycle such as thiophene (compound **6e**), the least reactivity toward binding with the protein receptor was observed.

**TABLE 3:** MST binding assay results of MDM2.

#	Code	Chemical structures	*K*_*D*_ (µM)
1	**6a**	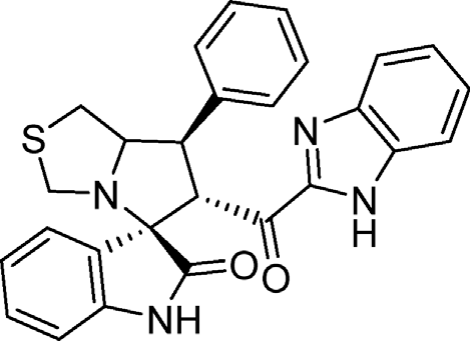	87.7
2	**6b**	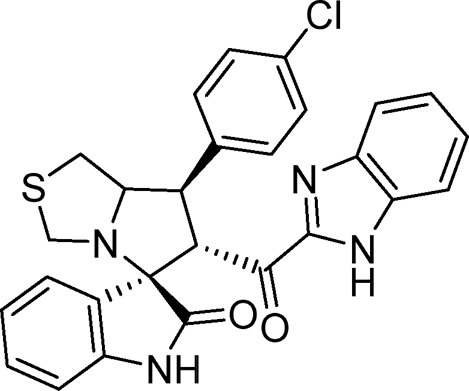	38.2
3	**6d**	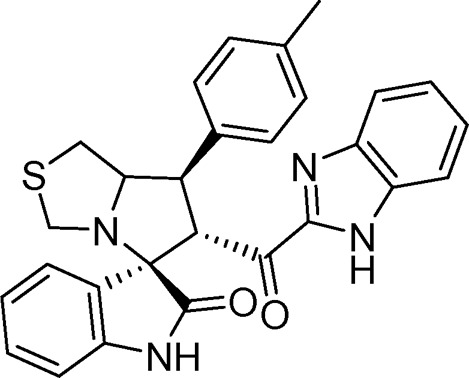	17.4
4	**6e**	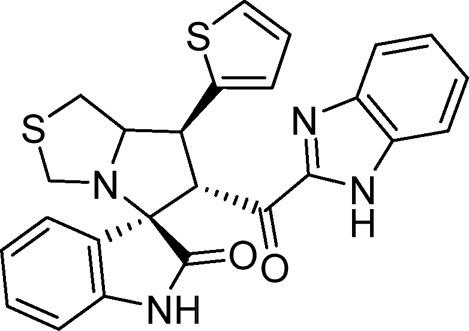	113
5	**6h**	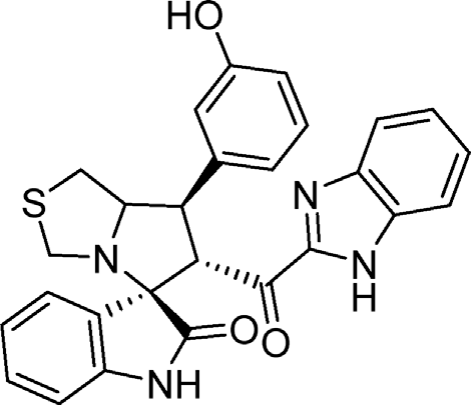	6.68
6	**6i**	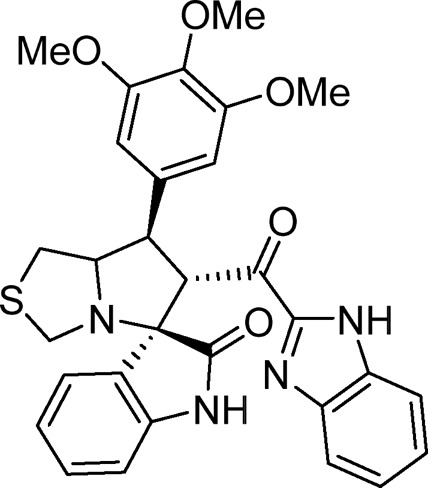	21
7	**6l**	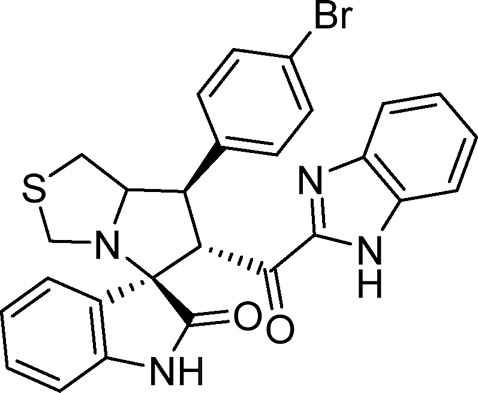	49.9

### 2.4 Molecular docking study

**FIGURE 10:**
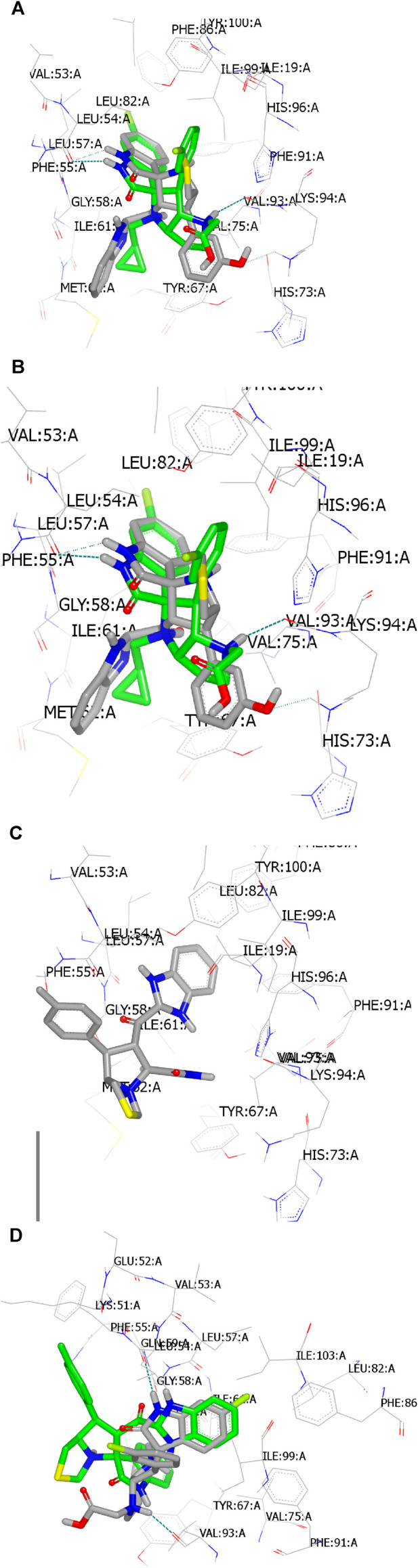
Snap shot visualization of compounds docked with PDB: ID 5law as represented by Vida: **(A)** standard ligand re docked with its co-crystalized coordinate; **(B)** compound **6h** showed HB interaction with Leu:54AA, and with Lys:94AA (green dotted line); **(C)** compound **6d** docked with the receptor through formation of hydrophobic-hydrophobic interactions; **(D)** compound **6d** (green) in comparison with standard ligand **6SJ** (grey).

The designed compounds (**6a–l**) and standard co-crystallized spirooxindole compounds were docked with the MDM2 protein, which was retrieved from the Protein Data Bank (PDB code: 5law) (Gollner et al., 2016a). The docking protocol was performed using OpenEye scientific software (Docking, 2016). To validate the docking protocol, the standard ligand **6SJ** was re-docked with the receptor. The standard ligand **6SJ** exhibited the same binding mode and pose as its co-crystallized complex (Gollner et al., 2016a), completely overlaying each other, as shown in Figure 10A. It formed HBs with Leu:54AA through the NH of the indole and with Lys:94AA through the protonated nitrogen of the pyrrolidine amino acid. Compound **6h** exhibited high similarity in binding pose and mode with the co-crystallized ligand (Figure 10B). It interacts with the receptor through formation of HBs with Leu:54AA through the NH of the indole, and with Lys:94AA through phenolic functionality. Figure 10C shows the deposition of compound **6d** inside the active receptor domains. The compound interacted with its receptor via hydrophobic interactions. Figure 10D shows the docking pose and mode of compound **6d** compared with those of the standard ligand **6SJ**. The benzimidazole ring of compound **6d was** overlaid with the indole moiety of the standard ligand **6SJ**. However, the remaining features of both compounds were oriented in different directions.

### 2.5 Shape alignment and ROCS analysis

The rapid overlay chemical similarity (ROCS) approach was used to determine compound similarity based on 3D structures and automated crafted descriptors using OpenEye software. Shape similarity was determined using Tanimoto scores. The Tanimoto Combo (maximum value = 2) is the sum of the shape (maximum value = 1) and color (maximum value = 1) Tanimoto scores. Considering **6SJ**, compound score was not correlated to activity. Compounds **6d** and **6h** had TC scores of 0.83 and 0.81, respectively, as shown in Table 4. The results indicated that our scaffolds could resemble another target or have unique chemometric characteristics.

**TABLE 4:** Tanimto combo scores of designed compounds using **6SJ** standard as ShapeQuery.

Vida name	Tanimoto combo	Shape tanimoto	Color tanimoto
**6sj law standard**	2	1	1
**6n**	0.902	0.658	0.244
**6j**	0.882	0.663	0.219
**6e**	0.843	0.634	0.209
**6m**	0.835	0.626	0.209
**6d**	0.834	0.625	0.209
**6h**	0.819	0.627	0.192
**6a**	0.816	0.598	0.218
**6k**	0.806	0.598	0.209
**6b**	0.805	0.587	0.218
**6g**	0.799	0.580	0.218
**6l**	0.797	0.588	0.209
**6c**	0.789	0.580	0.209
**6i**	0.710	0.535	0.175

**FIGURE 11:**
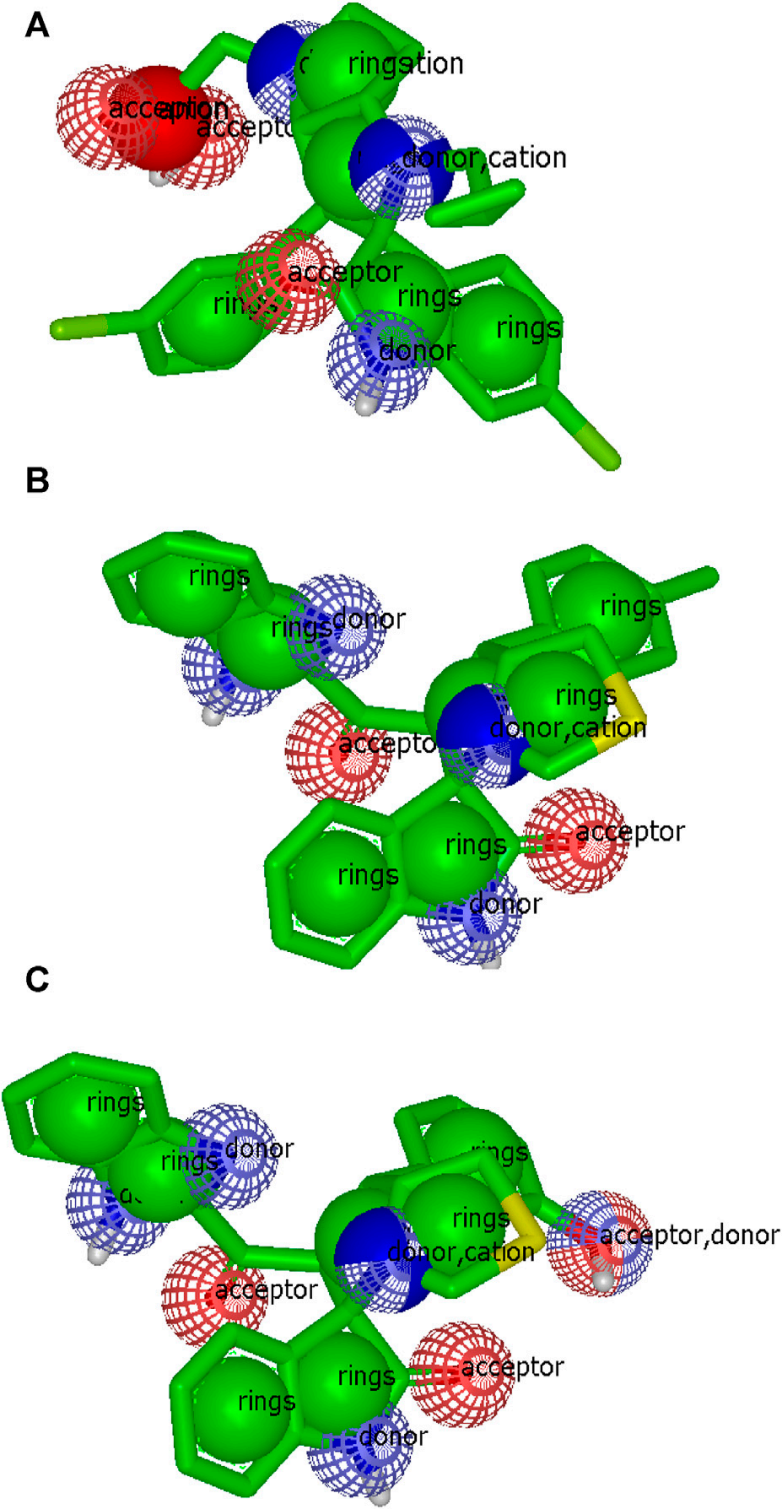
Topology display of color atoms and shape atoms. **(A)** standard **6SJ** (5 rings, 3 acceptors, 3 donors, 2 cations, 1 anion); **(B)** compound **6d** (7 rings, 2 acceptors, 4 donors, 1 cation; **(C)** compound **6h** (7 rings, 3 acceptors, 5 donors, 1 cation).

For compounds **6d** and **6h**, the color and shape atoms (generated by the ROCS application, OpenEye software) are shown in Figures 11A–C compared with the standard ligand (**6SJ**).

The most important descriptors for compounds **6d** and **6h** are presented in Table 5, compared with the **6SJ** standard. Both compounds showed values similar to the standard; however, the number of rotatable bonds was three in compounds **6d** and **6h** and five in **6SJ**. These results highlight the importance of the number of rotatable bonds, as previously reported (Abdelhady et al., 2022).

**TABLE 5:** Selected physiochemical properties of compounds **6d,h** and standard **6SJ** as generated by OpenEye software.

Compound	Molecular weight	LogP	PSA	No. of rotatable bond	No. of heavy atom	No. of donor	No. of acceptor
**6SJ**	488	3.19	75	5	33	4	6
**6d**	483	2.20	74	3	35	4	6
**6h**	485	3.19	94	3	35	5	7

## 3 Structure activity relationship

For MDM2 binding, compound **6h** showed stronger binding activity than **6d, 6i, 6b, 6l, 6a,** and **6e**. The electronic positions of the substituents on the phenyl rings of the benzaldehyde derivatives contributed in both the electronic and geometric properties of the final scaffolds. Compound **6h** contained a phenolic OH group at the meta position and formed a HB with Lys:94AA in the receptor. Furthermore, compound **6h** showed a unique orientation inside the receptor; none of the compounds overlapped with it. Compound **6d,** with a mildly activating group (Me) at the para position, showed low Logp and PSA values (Table 5). The PSA level was similar to that of **6SJ**. Compounds with electron-withdrawing groups, namely, *p*-Cl and *p*-Br, and thiophene moieties, exhibited lower activity, probably due to the electronic effect of the compound topology. The meta-position with formation of an extra HB was preferable for inhibitory activity (SI, Supplementary Figure S10).

### 3.1 Experimental

#### 3.1.1 General

“All chemicals were purchased from Aldrich, Sigma-Aldrich, and Fluka, and were used without further purification unless otherwise indicated. All melting points were measured using a Gallenkamp melting point apparatus in open glass capillaries and were uncorrected. The crude products were purified using silica gel through column chromatography (100–200 mesh). IR spectra were recorded as KBr pellets using a Nicolet 6700 FT-IR spectrophotometer. NMR spectra were recorded using a Varian Mercury Jeol-400 NMR spectrometer. ^1^H NMR (400 MHz) and ^13^C NMR (100 MHz) spectroscopy were performed in either deuterated dimethyl sulfoxide (DMSO-*d*_6_) or deuterated chloroform (CDCl_3_) (SI; Supplementary Figures S11–23). Chemical shifts (*δ*) are reported in ppm; coupling constant *J is* given in Hz. Elemental analysis was performed using an Elmer 2400 Elemental Analyzer in CHN mode. Chalcones **3a–n** was prepared as reported previously by (Alshahrani et al., 2023)”.

#### 3.1.2 General procedure for synthesis of spiro compounds (6a–n)

A mixture of chalcone derivative **3a–n** (0.5 mmol), thiazolidine-4-carboxylic acid (66.59 mg, 0.5 mmol), and isatin (73.57 mg, 0.5 mmol) in methanol (20 ml) was stirred at 60–65^o^C in an oil bath for 2–3 h. The final spirooxindole-based benzimidazole was separated using either by filtration if the final product precipitated or by column chromatography ((Silica Gel 60, granulometry 0.015–0.040 mm, Merck); eluent: EtOAc/nHexane gradient polarity according to the specified compound starting from 30:70 until 50:50).

#### 3.1.3 (3*S*,6′*R*)-6'-(1*H*-benzo [*d*]imidazole-2-carbonyl)-7′-phenyl-3′,6′,7′,7a′-tetrahydro-1′H-spiro [indoline-3.5′-pyrrolo [1,2-*c*]thiazol]-2-one (6a)

White solid; yield (89%); m.p: 240°C–242°C; IR (KBr, cm^−1^): 3432 (NH), 3276 (NH), 3094 (CH), 2934 (CH), 1732 (CO), 1684 (CO); ^1^H-NMR (400 MHz, DMSO-*d*_6_): *δ* 12.95 (1H, s, NH), 10.39 (1H, s, NH), 7.69 (1H, d, *J* = 8 Hz, ArH), 7.50 (2H, d, *J* = 8 Hz, ArH), 7.38–7.18 (7H, m, ArH), 6.99 (1H, t, *J* = 8 Hz, ArH), 6.84 (1H, t, *J* = 8 Hz, ArH), 6.44 (1H, d, *J* = 8 Hz, ArH), 5.17 (1H, d, *J* = 11.2 Hz, CHCO), 4.26 (1H, q, *J* = 8 Hz, CHN), 3.94 (1H, t, *J* = 10.2 Hz, CHPh), 3.68 (1H, d, *J* = 8.8 Hz), 3.40 (1H, d, *J* = 8.4 Hz), 3.18 (2H, d, *J* = 5.2 Hz), 2.98 (2 H, dd, *J* = 19.2, 5.3Hz); ^13^C-NMR (101 MHz, DMSO-*d*_6_): *δ* 190.05, 178.85, 147.94, 143.19, 142.94, 140.21, 135.11, 129.41, 128.55, 127.64, 124.31, 122.07, 113.19, 109.89, 74.51, 72.44, 63.53, 51.82, 50.08, 35.28; Anal. for C_27_H_22_N_4_O_2_S; calcd: C, 69.51; H, 4.75; N, 12.01; O, 6.86; S, 6.87; Found: C, 69.49; H, 4.77; N, 12.04; O, 6.82; S, 6.88.

#### 3.1.4 (3*S*,6′*R*)-6'-(1*H*-Benzo [*d*]imidazole-2-carbonyl)-7'-(4-chlorophenyl)-3′,6′,7′,7a′-tetrahydro-1′*H*-spiro [indoline-3.5′-pyrrolo [1,2-*c*]thiazol]-2-one (6b)

Pale yellow solid; yield (57%); m.p.: 210–212^o^C; IR (KBr, cm^−1^): 3434 (NH), 3277 (NH), 3093 (CH), 2929 (CH), 1729 (CO), 1682 (CO); ^1^H-NMR (400 MHz, DMSO-*d*_6_): *δ* 12.96 (1H, s, NH), 10.41 (1H, s, NH), 7.54 (3H, d, *J* = 8 Hz, ArH), 7.41 (3H, d, *J* = 8 Hz, ArH), 7.23 (3H, d, *J* = 7.3 Hz, ArH), 6.98 (1H, t, *J* = 7.6 Hz, ArH), 6.82 (1H, t, *J* = 7.6 Hz, ArH), 6.44 (1H, d, *J* = 7.8 Hz, ArH), 5.12 (1H, d, *J* = 11 Hz, CHCO), 4.23 (1H, q, *J* = 8 Hz, CHN), 3.95 (1H, t, *J* = 10 Hz, CHPh), 3.67 (1H, d, *J* = 8.8 Hz), 3.39 (2H, d, *J* = 8.8 Hz), 2.98 (2H, m); ^13^C-NMR (101 MHz, DMSO-*d*_6_): *δ* 189.91, 178.79, 147.91, 143.23, 139.26, 132.41, 130.42, 129.30, 124.23, 121.36, 109.96, 74.32, 72.35, 63.16, 49.17, 35.10, 27.77; Anal. for C_27_H_21_ClN_4_O_2_S; calcd: C, 64.73; H, 4.22; Cl, 7.08; N, 11.18; O, 6.39; S, 6.40; Found: C, 64.69; H, 4.27; Cl, 7.06; N, 11.20; O, 6.35; S, 6.43.

#### 3.1.5 (3*S*,6′*R*)-6'-(1*H*-Benzo [*d*]imidazole-2-carbonyl)-7'-(4-(trifluoromethyl) phenyl)-3′,6′,7′,7a′-tetrahydro-1′*H*-spiro [indoline-3.5′-pyrrolo [1,2-*c*]thiazol]-2-one (6c)

Yellow solid; yield (13.5%); m.p.:185–187^o^C; IR (KBr, cm^−1^): 3429 (NH), 3282 (NH), 3099 (CH), 2927 (CH), 1724 (CO), 1686 (CO); ^1^H-NMR (CDCl_3_, 400 MHz): *δ* 12.90 (1H, s, NH), 10.42 (1H, s, NH), 7.45 (2H, d, *J* = 8 Hz, ArH), 7.23 (4H, d, *J* = 8 Hz, ArH), 7.08 (1H, d, *J* = 8, ArH), 6.98 (1H, t, *J* = 7.7 Hz, ArH), 6.90 (1H, t, *J* = 7.7 Hz, ArH), 6.84 (1H, d, *J* = 7.3 Hz, ArH), 6.45 (2H, t, *J* = Hz, ArH), 5.16 (1H, d, *J* = Hz, CHCO), 4.24 (1H, q, *J* = Hz, CHN), 4.06 (1H, t, *J* = Hz, CHPh), 3.68 (2H, d, *J* = 8.8 Hz), 3.25 (1H, d, *J* = 12 Hz), 3.00 (2H, m); Anal. for C_28_H_21_F_3_N_4_O_2_S; calcd: C, 62.91; H, 3.96; F, 10.66; N, 10.48; O, 5.99; S, 6.00; Found: C, 62.96; H, 3.92; F, 10.67; N, 10.42; O, 6.01; S, 6.02.

#### 3.1.6 (3*S*,6′*R*)-6'-(1*H*-Benzo [*d*]imidazole-2-carbonyl)-7'-(p-tolyl)-3′,6′,7′,7a′-tetrahydro-1′*H*-spiro [indoline-3.5′-pyrrolo [1,2-*c*]thiazol]-2-one (6d)

White solid; yield (66%); m.p.:228–230^o^C; IR (KBr, cm^−1^): 3649 (NH), 3277 (NH), 3087(CH), 2926 (CH), 1727 (CO), 1690 (CO); ^1^H-NMR (400 MHz, DMSO-*d*_6_): *δ* 12.94 (1H, s, NH), 10.37 (1H, s, NH), 7.69 (1H, d, *J* = Hz, ArH), 7.38–7.20 (6H, m, ArH), 7.15 (2H, d, *J* = Hz ArH), 6.99 (1H, t, *J* = 7.6 Hz, ArH), 6.84 (1H, t, *J* = 7.6 Hz, ArH), 6.44 (1H, d, *J* = 7.8 Hz, ArH), 5.15 (1H, d, *J* = 11.3 Hz, CHCO), 4.20 (1H, q, *J* = 8 Hz, CHN), 3.88 (1H, t, *J* = 10.6 Hz, CHPh), 3.67 (1H, d, *J* = 8.8 Hz), 3.39 (2H, d, *J* = 8.8 Hz), 3.18 (1H, d, *J* = 5.1 Hz), 2.96 (2H, m), 2.23 (3H, s, CH_3_); ^13^C-NMR (101 MHz, DMSO-*d*_6_): *δ* 190.08, 178.84, 147.98, 143.16, 142.96, 137.03, 136.93, 135.10, 130.01, 128.37, 127.49, 126.34, 124.35, 123.43, 122.00, 121.94, 121.37, 113.28, 109.93, 74.49, 72.44, 63.24, 51.84, 49.89, 35.25, 21.23; Anal. for C_28_H_24_N_4_O_2_S; calcd: C, 69.98; H, 5.03; N, 11.66; O, 6.66; S, 6.67; Found: C, 69.95; H, 5.06; N, 11.68; O, 6.63; S, 6.68.

#### 3.1.7 (3*S*,6′*R*)-6'-(1*H*-Benzo [*d*]imidazole-2-carbonyl)-7'-(thiophen-2-yl)-3′,6′,7′,7a′-tetrahydro-1′*H*-spiro [indoline-3.5′-pyrrolo [1,2-*c*]thiazol]-2-one (6e)

White solid; yield (76%); m.p.:240–242^o^C; IR (KBr, cm^−1^): 3624 (NH), 3258 (NH), 3091(CH), 2927 (CH), 1728 (CO), 1689 (CO); ^1^H-NMR (400 MHz, DMSO-*d*_6_): *δ* 13.02 (1H, s, NH), 10.35 (1H, s, NH), 7.71 (1H, d, *J* = 8 Hz, ArH), 7.40 (1H, d, *J* = 5.1 Hz, CHS), 7.37–7.21 (4H, m, ArH), 7.12 (1H, d, *J* = 3.5 Hz, ArH), 7.04–6.94 (2H, m, ArH), 6.84 (1H, t, *J* = 7.5 Hz, ArH), 6.44 (1H, d, *J* = 7.5 Hz, ArH), 5.07 (1H, d, *J* = 10.3 Hz, CHCO), 4.25–4.18 (2H, m), 3.68 (1H, d, *J* = 9.3 Hz), 3.39 (1H, d, *J* = 9.1 Hz), 3.21–3.01 (4H, m); ^13^C-NMR (101 MHz, DMSO-*d*_6_): *δ* 189.66, 178.54, 147.84, 143.13, 143.00, 142.76, 135.15, 130.26, 127.66, 126.00, 124.01, 113.42, 109.93, 74.31, 72.66, 63.78, 52.25, 45.39, 35.45; Anal. for C_25_H_20_N_4_O_2_S_2_; calcd: C, 63.54; H, 4.27; N, 11.86; O, 6.77; S, 13.57; Found: C, 63.49; H, 4.24; N, 11.84; O, 6.80; S, 13.63.

#### 3.1.8 (3*S*,6′*R*)-6'-(1*H*-Benzo [*d*]imidazole-2-carbonyl)-7'-(4-fluorophenyl)-3′,6′,7′,7a′-tetrahydro-1′*H*-spiro [indoline-3.5′-pyrrolo [1,2-*c*]thiazol]-2-one (6f)

White solid; yield (31%); m.p.:233–235^o^C; IR (KBr, cm^−1^): 3431 (NH), 3268 (NH), 3096 (CH), 2960 (CH), 1732 (CO), 1684 (CO); ^1^H-NMR (400 MHz, DMSO-*d*_6_): *δ* 12.95 (1H, s, NH), 10.39 (1H, s, NH), 7.68 (1H, d, *J* = 8 Hz, ArH), 7.54 (2H, t, *J* = 6.8 Hz, ArH), 7.36–7.14 (6H, m, ArH), 6.98 (1H, t, *J* = 7.7 Hz, ArH), 6.83 (1H, t, *J* = 7.7 Hz, ArH), 6.43 (1H, d, *J* = 7.4 Hz, ArH), 5.11 (1H, d, *J* = 11 Hz, CHCO), 4.20 (1H, q, J = 9.6 Hz, CHN), 3.95 (1H, t, *J* = 10.3 Hz, CHPh), 3.67 (1H, d, *J* = 8.8 Hz), 3.40 (1H, d, *J* = 12 Hz), 2.98 (2H, m); ^13^C-NMR (101 MHz, DMSO-*d*_6_): *δ* 189.97, 178.79, 169.71, 147.90, 143.21, 135.10, 130.23, 124.24, 72.33; Anal. for C_27_H_21_FN_4_O_2_S; calcd C, 66.93; H, 4.37; F, 3.92; N, 11.56; O, 6.60; S, 6.62; Found: C, 66.97; H, 4.34; F, 3.94; N, 11.53; O, 6.63; S, 6.59.

#### 3.1.9 (3*S*,6′*R*)-6'-(1*H*-Benzo [*d*]imidazole-2-carbonyl)-7'-(2,4-dichlorophenyl)-3′,6′,7′,7a′-tetrahydro-1′*H*-spiro [indoline-3.5′-pyrrolo [1,2-*c*]thiazol]-2-one (6g)

White solid; yield (38%); m.p.:235–237^o^C; IR (KBr, cm^−1^): 3436 (NH), 3281 (NH), 3090 (CH), 2925 (CH), 1730 (CO), 1685 (CO); ^1^H-NMR (400 MHz, DMSO-*d*_6_): *δ* 12.96 (1H, s, NH), 10.44 (1H, s, NH), 7.85 (1H, d, *J* = 8.5 Hz, ArH), 7.69 (1H, d, *J* = 8 Hz, ArH), 7.63 (1H, s, ArH), 7.51 (1H, d, *J* = 8 Hz, ArH), 7.34–7.14 (4H, m, ArH), 6.97 (1H, t, *J* = 7.6 Hz, ArH), 6.82 (1H, t, *J* = 7.6 Hz, ArH), 6.43 (1H, t, *J* = 7.6 Hz, ArH), 5.11 (1H, d, *J* = 10 Hz, CHCO), 4.57 (1H, t, *J* = 9.1 Hz, CHPh), 4.14 (1H, q, *J* = 8 Hz, CHN), 3.72 (1H, d, *J* = 8.8 Hz), 3.42 (1H, d, *J* = 8.8 Hz), 3.09 (1H, dd, *J* = 11, 5.9 Hz), 2.92 (1H, dd, *J* = 11, 4.4 Hz); ^13^C-NMR (101 MHz, DMSO-*d*_6_): *δ* 189.72, 178.88, 147.75, 143.42, 142.88, 137.30, 134.99, 132.88, 130.31, 129.50, 128.68, 127.37, 126.42, 123.69, 121.85, 121.39, 113.26, 109.96, 75.13, 72.88, 62.72, 52.52, 44.70, 35.44; Anal. for C_27_H_20_Cl_2_N_4_O_2_S; calcd C, 60.56; H, 3.76; Cl, 13.24; N, 10.46; O, 5.98; S, 5.99; Found: C, 60.50; H, 3.71; Cl, 13.34; N, 10.41; O, 6.03; S, 6.01.

#### 3.1.10 (3*S*,6′*R*)-6'-(1*H*-Benzo [*d*]imidazole-2-carbonyl)-7'-(3-hydroxyphenyl)-3′,6′,7′,7a′-tetrahydro-1′*H*-spiro [indoline-3.5′-pyrrolo [1,2-*c*]thiazol]-2-one (6h)

White solid; yield (82%); m.p.:205–207^o^C; IR (KBr, cm^−1^): 3626 (NH), 3257 (OH), 2931 (CH), 1726 (CO), 1688 (CO); ^1^H-NMR (400 MHz, DMSO-*d*_6_): *δ* 12.94 (1H, s, NH), 10.36 (1H, s, NH), 9.46 (1H, s, OH), 7.69 (1H, d, *J* = 8 Hz, ArH), 7.33 (1H, d, *J* = 8 Hz, ArH), 7.28 (1H, t, *J* = 8 Hz, ArH), 7.21 (2H, d, *J* = 6.7 Hz, ArH), 7.13 (1H, t, *J* = 8 Hz, ArH), 6.98 (1H, t, *J* = 7.7 Hz, ArH), 6.89 (2H, d, *J* = 7.3 Hz, ArH), 6.83 (1H, t, *J* = 7.3 Hz, ArH), 6.62 (1H, d, *J* = 8 Hz, ArH), 6.43 (1H, d, *J* = 8 Hz, ArH), 5.14 (1H, d, *J* = 11.7 Hz, CHCO), 4.18 (1H, q, *J* = 8 Hz, CHN), 3.83 (1H, t, *J* = 10.3 Hz, CHPh), 3.66 (1H, d, *J* = 8.8 Hz), 3.02 (1H, dd, *J* = 11.0, 5.9 Hz), 2.92 (1H, dd, *J* = 10.6, 4.8 Hz); ^13^C-NMR (101 MHz, DMSO-*d*_6_): *δ* 190.06, 178.84, 158.20, 147.97, 143.14, 142.94, 141.56, 135.11, 130.12, 127.50, 124.36, 123.55, 121.88, 119.21, 114.98, 109.83, 72.32, 63.26, 51.59, 49.90, 35.27; Anal. for C_27_H_22_N_4_O_3_S; calcd C, 67.20; H, 4.60; N, 11.61; O, 9.95; S, 6.64; Found: C, 67.15; H, 4.64; N, 11.65; O, 9.91; S, 6.65.

#### 3.1.11 (3*S*,6′*R*)-6'-(1*H*-Benzo [d]imidazole-2-carbonyl)-7'-(3,4,5-trimethoxyphenyl)-3′,6′,7′,7a′-tetrahydro-1′*H*-spiro [indoline-3.5′-pyrrolo [1,2-*c*]thiazol]-2-one (6i)

White solid; yield (70%); m.p.:245–247^o^C; IR (KBr, cm^−1^): 3430 (NH), 3269 (NH), 3093 (CH), 2996 (CH), 1722 (CO), 1683 (CO); ^1^H-NMR (400 MHz, DMSO-*d*_6_): *δ* 12.95 (1H, s, NH), 10.35 (1H, s, NH), 7.70 (1H, d, *J* = 8 Hz, ArH), 7.35–7.21 (5H, m, ArH), 7.00 (1H, t, *J* = 7.6 Hz, ArH), 6.85 (2H, d, *J* = 7.7 Hz, ArH), 6.46 (1H, d, *J* = 7.8 Hz, ArH), 5.13 (1H, d, *J* = 11 Hz, CHCO), 4.20 (1H, m, CHN), 3.88 (1H, t, *J* = 9.9 Hz, CHPh), 3.79 (6H, s, OCH_3_), 3.69 (1H, d, *J* = 8.8 Hz), 3.60 (3H, s, OCH_3_), 3.32 (2H, d, *J* = 3 Hz), 3.03 (2H, m); ^13^C-NMR (101 MHz, DMSO-*d*_6_): *δ* 190.06, 178.90, 153.56, 148.00, 143.13, 142.97, 137.12, 135.94, 135.08, 130.17, 127.79, 126.43, 124.10, 123.64, 121.88, 121.25, 109.89, 105.71, 74.66, 73.00, 62.56, 60.48, 56.47, 52.43, 50.62, 35.70; Anal. for C_30_H_28_N_4_O_5_S; calcd C, 64.73; H, 5.07; N, 10.07; O, 14.37; S, 5.76; Found: C, 64.69; H, 5.10; N, 10.11; O, 14.33; S, 5.77.

#### 3.1.12 (3*S*,6′*R*)-6'-(1*H*-Benzo [d]imidazole-2-carbonyl)-7'-(2-hydroxyphenyl)-3′,6′,7′,7a′-tetrahydro-1′*H*-spiro [indoline-3.5′-pyrrolo [1,2-*c*]thiazol]-2-one (6j)

Pale yellow solid; yield (25%); m.p.:186–188^o^C; IR (KBr, cm^−1^): 3311 (OH), 3063 (CH), 2925 (CH), 1716 (CO), 1667 (CO); ^1^H-NMR (400 MHz, DMSO-*d*_6_): *δ* 12.87 (1H, s, NH), 10.28 (1H, s, NH), 9.63 (1H, s, OH), 7.70 (1H, d, *J* = 8 Hz, ArH), 7.41 (1H, d, *J* = 8 Hz, ArH), 7.34–7.22 (4H, m, ArH), 7.05–6.98 (2H, m, ArH), 6.86–6.76 (3H, m, ArH), 6.44 (1H, d, *J* = 3 Hz, ArH), 5.40 (1H, d, *J* = 11.0 Hz, CHCO), 4.27–4.22 (1H, m, CHN), 3.68 (1H, t, *J* = 9.5 Hz, CHPh), 3.55–3.48 (2H, m), 3.33 (2H, d, *J* = 7.3 Hz); ^13^C-NMR (101 MHz, DMSO-*d*_6_): *δ* 190.40, 178.94, 156.17, 148.15, 143.14, 129.74, 129.31, 129.09, 128.33, 125.87, 124.22, 121.45, 119.85, 115.99, 109.78, 73.56, 73.05, 60.40, 52.69, 44.80, 35.87, 28.42; Anal. for C_27_H_22_N_4_O_3_S; calcd C, 67.20; H, 4.60; N, 11.61; O, 9.95; S, 6.64; Found: C, 67.26; H, 4.66; N, 11.54; O, 9.89; S, 6.65.

#### 3.1.13 (3*S*,6′*R*)-6'-(1*H*-Benzo [d]imidazole-2-carbonyl)-7'-(4-(dimethylamino)phenyl)-3′,6′,7′,7a′-tetrahydro-1′*H*-spiro [indoline-3.5′-pyrrolo [1,2-*c*]thiazol]-2-one (6k)

Yellow solid; yield (45%); m.p.:178–180^o^C; IR (KBr, cm^−1^): 3434 (NH), 3275 (NH), 3094 (CH), 2929 (CH), 1729 (CO), 1682 (CO); ^1^H-NMR (400 MHz, DMSO-*d*_6_): *δ* 12.91 (1H, s, NH), 10.33 (1H, s, NH), 7.69 (1H, d, *J* = 8 Hz, ArH), 7.35–7.21 (6H, m, ArH), 6.99 (1H, t, *J* = 7.7 Hz, ArH), 6.84 (1H, t, *J* = 7.3 Hz, ArH), 6.69 (2H, d, *J* = 8 Hz, ArH), 6.45 (1H, d, *J* = 8 Hz, ArH), 5.10 (1H, d, *J* = 11.7 Hz, CHCO), 4.20–4.10 (1H, m, CHN), 3.78 (2H, t, *J* = 10.6 Hz, CHPh), 3.67 (2H, d, *J* = 9.5 Hz), 3.54 (3H, d, *J* = 3.7 Hz), 2.95 (2H, m), 2.82 (6H, s, NCH_3_); ^13^C-NMR (101 MHz, DMSO-*d*_6_): *δ* 190.23, 178.95, 150.25, 148.05, 143.04, 142.96, 135.07, 130.10, 128.96, 127.69, 127.02, 126.39, 124.37, 123.58, 121.93, 121.34, 113.35, 109.86, 74.50, 72.63, 63.05, 55.45, 52.21, 49.73, 40.70, 35.44; Anal. for C_29_H_27_N_5_O_2_S; calcd C, 68.35; H, 5.34; N, 13.74; O, 6.28; S, 6.29; Found: C, 68.45; H, 5.39; N, 13.54; O, 6.27; S, 6.26.

#### 3.1.14 (3*S*,6′*R*)-6'-(1*H*-Benzo [d]imidazole-2-carbonyl)-7'-(4-bromophenyl)-3′,6′,7′,7a′-tetrahydro-1′*H*-spiro [indoline-3.5′-pyrrolo [1,2-*c*]thiazol]-2-one (6l)

White solid; yield (80%); m.p.:172–174^o^C; IR (KBr, cm^−1^): 3625 (NH), 3422 (NH), 3088 (CH), 2913 (CH), 1725 (CO), 1682 (CO); ^1^H-NMR (400 MHz, DMSO-*d*_6_): *δ* 12.95 (1H, s, NH), 10.40 (1H, s, NH), 7.68 (1H, d, *J* = 8 Hz, ArH), 7.55 (2H, d, *J* = 8 Hz, ArH), 7.47 (2H, d, J = 8.4 Hz, ArH), 7.36–7.19 (4H, m, ArH), 6.98 (1H, t, *J* = 7.6 Hz, ArH), 6.82 (1H, t, *J* = 7.6 Hz, ArH), 6.43 (1H, d, *J* = 7.6 Hz, ArH), 5.10 (1H, d, *J* = 10.9 Hz, CHCO), 4.24–4.14 (1H, m, CHN), 3.93 (1H, t, *J* = 8 Hz, CHPh), 3.67 (1H, d, *J* = 8.8 Hz), 3.38 (2H, d, *J* = 8.8 Hz), 3.04–2.92 (2H, m); ^13^C- NMR (101 MHz, DMSO-*d*_6_): *δ* 189.88, 178.76, 147.85, 143.23, 142.92, 139.67, 135.10, 132.27, 130.51, 130.10, 127.41, 124.21, 123.99, 121.47, 120.91, 113.25, 109.86, 74.15, 72.34, 62.95, 51.65, 49.41, 33.89; Anal. for C_27_H_21_BrN_4_O_2_S; calcd C, 59.45; H, 3.88; Br, 14.65; N, 10.27; O, 5.87; S, 5.88; Found: C, 59.51; H, 3.81; Br, 14.55; N, 10.31; O, 5.97; S, 5.85.

#### 3.1.15 (3*S*,6′*R*)-6'-(1*H*-Benzo [d]imidazole-2-carbonyl)-7'-(3-fluorophenyl)-3′,6′,7′,7a′-tetrahydro-1′*H*-spiro [indoline-3.5′-pyrrolo [1,2-*c*]thiazol]-2-one (6m)

White solid; yield (30%); m.p.:224–226^o^C; IR (KBr, cm^−1^): 3625 (NH), 3422 (NH), 3088 (CH), 2913 (CH), 1725 (CO), 1682 (CO); ^1^H-NMR (400 MHz, DMSO-*d*_6_) *δ* 12.97 (s, 1H, NH), 10.41 (s, 1H, NH), 7.68 (d, *J* = 8.1 Hz, 1H, ArH), 7.46–7.19 (m, 7H, ArH), 7.09 (t, *J* = 8.4 Hz, 1H, ArH), 6.98 (t, *J* = 7.7 Hz, 1H, ArH), 6.82 (t, *J* = 7.7 Hz, 1H, ArH), 6.43 (d, *J* = 7.3 Hz, 1H, ArH), 5.11 (d, *J* = 11.0 Hz, 1H, COCH), 4.22 (dt, *J* = 11.0, 5.5 Hz, 1H, CHN), 3.98 (t, *J* = 10.3 Hz, 1H, CHPh), 3.67 (d, *J* = 8.8 Hz, 1H), 3.38 (d, *J* = 5.1 Hz, 1H), 3.04 (dd, *J* = 11.0, 5.9 Hz, 1H), 2.96 (dd, *J* = 11.0, 5.9 Hz, 1H); ^13^C-NMR (101 MHz, DMSO-*d*_6_) *δ* 189.88, 178.79, 147.86, 143.36, 143.23, 142.91, 135.10, 131.42, 131.33, 130.20, 127.44, 126.44, 124.57, 124.17, 123.60, 121.90, 121.38, 115.37, 114.54, 113.27, 109.90, 74.36, 72.39, 63.35, 51.67, 49.41, 35.17; Anal. for C_27_H_21_FN_4_O_2_S; calcd C, 66.93; H, 4.37; F, 3.92; N, 11.56; O, 6.60; S, 6.62; Found: C, 66.84; H, 4.44; F, 3.95; N, 11.49; O, 6.60; S, 6.68.

#### 3.1.16 (3*S*,6′*R*)-6'-(1*H*-Benzo [d]imidazole-2-carbonyl)-7'-(furan-2-yl)-3′,6′,7′,7a′-tetrahydro-1′*H*-spiro [indoline-3.5′-pyrrolo [1,2-*c*]thiazol]-2-one (6n)

White solid; yield (29%); m.p.:240–242^o^C; IR (KBr, cm^−1^): 3435 (NH), 3256 (NH), 3090 (CH), 2928 (CH), 1729 (CO), 1687 (CO); ^1^H-NMR (DMSO-*d*_6_, 400 MHz): *δ* 13.01 (1H, s, NH), 10.33 (1H, s, NH), 7.71 (1H, d, *J* = 8 Hz, ArH), 7.58 (1H, d, *J* = 4 Hz, ArH), 7.38–7.18 (4H, m, ArH), 6.99 (1H, t, *J* = 7.7 Hz, ArH), 6.83 (1H, t, *J* = 7.7 Hz, ArH), 6.42 (1H, d, *J* = 8 Hz, ArH), 6.40–6.31 (2H, m), 5.14 (1H, d, *J* = 11 Hz, CHCO), 4.20 (1H, m, CHN), 4.02 (1H, t, *J* = 8 Hz, CHPh), 3.66 (1H, d, *J* = 9.5 Hz), 3.37 (2H, d, *J* = 8.8 Hz), 3.12 (2H, m); ^13^C-NMR (DMSO-*d*_6_, 100 MHz): *δ* 189.60, 178.55, 153.16, 147.78, 143.17, 142.99, 135.16, 126.57, 124.00, 110.01, 72.40; Anal. for C_25_H_20_N_4_O_3_S; calcd C, 65.77; H, 4.42; N, 12.27; O, 10.51; S, 7.02; Found: C, 65.73; H, 4.38; N, 12.31; O, 10.47; S, 7.11.

#### 3.1.3 Computational details

“The *ω*B97X-D (Chai and Head-Gordon, 2008) functional, together with the standard 6–311G (d,p) basis set (Warren et al., 1986) were used in this MEDT study. The TSs were characterized by the presence of only one imaginary frequency. IRC (Fukui, 1970) calculations were performed to establish a unique connection between the TSs and corresponding minima (Gonzalez and Schlegel, 1990; Gonzalez and Schlegel, 1991). Solvent effects of methanol were considered through full optimization of gas phase structures at the same computational level using the polarizable continuum model (PCM) (Tomasi and Persico, 1994; Simkin and Sheikhet, 1995) in the framework of the self-consistent reaction field (SCRF) (Cossi et al., 1996; Cancès et al., 1997; Barone et al., 1998). Values of *ω*B97X-D/6–311G (d,p) enthalpies, entropies, and Gibbs free energies in methanol were calculated using standard statistical thermodynamics (Warren et al., 1986) at 337.8 K and 1 atm, with PCM frequency calculations at the solvent optimized structures.

The GEDT (Domingo, 2014) values were computed using the equation GEDT(f) = Σq_f_, where q represents the natural charges (Reed et al., 1985; Reed et al., 1988) of the atoms belonging to one of the two frameworks (f) in the TS geometries. Global and local CDFT indices were calculated using the equations given in Domingo et al. (2016).

The Gaussian 16 program suite was used to perform the calculations. (Frisch et al. 2016) and ELF (Becke and Edgecombe, 1990) analyses of the *ω*B97X-D/6–311G (d,p) monodeterminantal wavefunctions were conducted using the TopMod (Noury et al., 1999) package with a cubical grid with a step size of 0.1 Bohr. AIM (Richard et al., 1982) calculations were performed using Multiwfn (Version 3.8) software (Lu and Chen, 2012). Molecular geometries and ELF basin attractors were visualized using GaussView (Serİn and Doğan Ulu, 2023)”.

#### 3.1.4 Molecular docking

The x-ray crystal structure coordinates of the angiotensin receptors were retrieved from PDB (PDB ID: 5law) (Gollner et al., 2016a) with their co-crystallized bound ligand and MDM2. The docking study was performed using OpenEye scientific software, version 2.2.5 (Santa Fe, NM, United States of America) (Gollner et al., 2016a). A virtual library of the synthesized compounds was used; their energies were minimized using the MMFF94 force field, followed by generation of multi-conformers using the OMEGA application. The library was compiled into one file using Omega software. The target proteins were retrieved from PDB, and the created receptor was operated using the OeDocking application. Both ligand and receptor input files were subjected to FRED in the molecular docking study. Multiple scoring functions were used to predict the energy profiles of the ligand–receptor complex. The Vida application was used for visualization. The dimensions for the created box of receptors were as follows. Box volume: 5544 A, dimensions: 21.00 A˚×18.00 A˚ ×14.67 A˚.

#### 3.1.5 Shape similarity and ROCS analysis

ROCS analysis was performed using OpenEye scientific software. The compound library was used as a database file. Both the query and database files were energy-minimized using Omega applications. The vROCS was used to run, analyze, and visualize the results. The ROCS application searches the database with a query to identify molecules with similar shapes and colors. The results were visualized using the Vida application. Compound conformers were scored based on the Gaussian overlap with the query. The best scoring parameters were the Tanimoto Combo scores (shape + color); the highest score indicated the best match with the query compound.

## 4 Conclusion

A new set of spirooxindole hybrids with a benzimidazole unit was designed, synthesized, and further assessed for anti-cancer reactivity. An MEDT study of the 32CA reaction of azomethine ylide (AY) **7a** with ethylene **3a** indicated that the reaction proceeded *via* a non-concerted *two-stage one-step* mechanism involving a highly asynchronous **TS-on** resulting from the nucleophilic attack of AY **7a** on the *β*-conjugated position of ethylene **3a**. Formation of two HBs at an earlier stage of the reaction accounted for the *ortho/endo* selectivities experimentally observed in these 32CA reactions. The anti-cancer reactivity results are promising; two compounds (**6a** and **6d)** were identified as most potent, safe, and as p53 activators in triple negative breast (MDA-MB 231) cancer cells to suppress expression, upregulating p21 expression by greater than three times and downregulating the expression of cyclin D and NF-kB. Moreover, the synthesized compounds showed high binding affinity for MDM2. Compound **6d** showed high similarity in binding mode to the co-crystallized standard ligand. Both compounds showed good physicochemical properties, which motivated us to investigate them further in preclinical studies.

## Data Availability

The original contributions presented in the study are included in the article/Supplementary Material, further inquiries can be directed to the corresponding author.
