# Supplementary material for: Activation of p53 signaling and regression of breast and prostate carcinoma cells by spirooxindole-benzimidazole small molecules
Source: Front Pharmacol. 2024 Apr 8;15:1358089. doi: 10.3389/fphar.2024.1358089 (PMC11033320; doi:10.3389/fphar.2024.1358089)
Supplement: Supplementary file 1 [file DataSheet1.docx]

**Supplementary Information**

Activation of p53 Signaling and Regression of Breast and Prostate Carcinoma Cells by Spirooxindole-Benzimidazole Small Molecules

Assem Barakat^1,*^, Saeed Alshahrani^1^, Abdullah Mohammed Al-Majid ^1^, Abdullah Saleh Alamary ^1^, Matti Haukka ^2^, Marwa M. Abu-Serie^3^, Alexander Dömling^4^, Luis R. Domingo^5^, Yaseen A. M. M. Elshaier^6^

1. Department of Chemistry, College of Science, King Saud University, P. O. Box 2455, Riyadh 11451, Saudi Arabia.
2. Department of Chemistry, University of Jyväskylä, P.O. Box 35, FI-40014 Jyväskylä, Finland.
3. Medical Biotechnology Department, Genetic Engineering and Biotechnology Research Institute, City of Scientific Research and Technological Applications (SRTA-City), Egypt.
4. Institute of Molecular and Translational Medicine, Faculty of Medicine and Dentistry and Czech Advanced Technology and Research Institute, Palackӯ University in Olomouc, Olomouc, Czech Republic.
5. Department of Organic Chemistry, University of Valencia, Dr. Moliner 50, 46100 Burjassot, Valencia, Spain.
6. Department of Organic and Medicinal Chemistry, Faculty of Pharmacy, University of Sadat City, Menoufiya 32958, Egypt.

***** Correspondence; E-mail: [ambarakat@ksu.edu.sa](mailto:ambarakat@ksu.edu.sa) (A.B.).

**General procedure for the synthesis of benzimidazolyl- chalcones (3a-n)**

The chosen aromatic aldehydes **2a-n** (3.125 mmol) was added to 2-acetylbenzimidazole **1** (500 mg, 3.125 mmol) in ethanol solution of sodium hydroxide (10% NaOH). The reaction mixture was subsequently stirred at room temperature overnight and neutralized with a solution of 30% acetic acid leading to a precipitate. It was filtered, dried to give compounds **3a-n**.

**Table S1:** Physical properties for the compounds **3a-n**.

| **Compounds 3a-n** | **Yield** | **m.p (^o^C)** | **Colour** |
| --- | --- | --- | --- |
| ****  **3a** | 80% | 197-199 | Pale yellow |
| ****  **3b** | 86% | 196-198 | Yellow |
|   **3c** | 62% | 209-211 | White |
| ****  **3d** | 72% | 208-210 | Pale yellow |
| ****  **3e** | 95% | 188-190 | Yellow |
| ****  **3f** | 98% | 189-191 | Yellow |
| ****  **3g** | 89% | 218-220 | Yellow |
| ****  **3h** | 66% | 217-219 | Yellow |
| ****  **3i** | 94% | 178-180 | Yellow |
| ****  **3j** | 65% | 187-189 | Yellow |
| ****  **3k** | 82% | 239-240 | Red |
| ****  **3l** | 89% | 210-212 | Yellow |
| ****  **3m** | 98% | 178-180 | Yellow |
| ****  **3n** | 92% | 190-192 | Yellow |

**X-Ray structure determinations**

The crystal of **6i** was immersed in cryo-oil, mounted in a loop, and measured at a temperature of 120 K. The X-ray diffraction data was collected on a Rigaku Oxford Diffraction Supernova diffractometer using Cu Kα radiation. The *CrysAlisPro^1^* software package was used for cell refinement and data reduction. A Multi-scan, absorption correction (*CrysAlisPro^1^*) was applied to the intensities before structure solution. Structure was solved by intrinsic phasing (*SHELXT^2^*) method. Structural refinement was carried out using *SHELXL^3^* software with *SHELXLE^4^* graphical user interface. The dichloromethane of crystallization was slightly disordered around two-fold rotation axis. Therefore, the carbon atom of the solvent molecule was restrained so that its U_ij_ components approximate to isotropic behavior. The NH hydrogen atoms were located from the difference Fourier map and refined isotropically. All other hydrogen atoms were positioned geometrically and constrained to ride on their parent atoms, with C-H = 0.95 -1.00 Å and U_iso_ = 1.2-1.5·U_eq_(parent atom). The crystallographic details are summarized in Table **S2**.

**Table S2.** Crystal Data.

|  | **6i** |
| --- | --- |
| CCDC | 2215719 |
| empirical formula | C_121_H_114_Cl_2_N_16_O_20_S_4_ |
| fw | 2311.42 |
| temp (K) | 120(2) |
| *λ*(Å) | 1.54184 |
| cryst syst | Monoclinic |
| space group | C2/c |
| *a* (Å) | 27.4596(3) |
| *b* (Å) | 13.5456(2) |
| *c* (Å) | 14.67290(10) |
| α(deg) |  |
| β (deg) | 93.9690(10) |
| γ(deg) |  |
| *V* (Å^3^) | 5444.59(11) |
| Z | 2 |
| *ρ*_calc_ (Mg/m^3^) | 1.410 |
| *μ*(Mo K*α*) (mm^-1^) | 1.916 |
| No. reflns. | 23304 |
| Unique reflns. | 5702 |
| Completeness to θ=67.684° | 100 % |
| GOOF (*F*^2^) | 1.044 |
| *R_int_* | 0.0240 |
| *R*_1_^a^ (*I* ≥ 2σ) | 0.0495 |
| *wR*_2_^b^ (*I* ≥ 2σ) | 0.1269 |

*^a^* *R_1_* = Σ||*F*_o_| – |*F*_c_||/Σ|*F*_o_|. *^b^wR*_2_ = {Σ[*w*(*F*_o_^2^ – *F*_c_^2^)^2^]/ Σ[*w*(*F*_o_^2^)^2^]}^1/2^

Table S3: Selected bond lengths [Å] and angles [^o^] for 6i.

| **Bond lengths [Å]** | **Bond angles [˚]** |
| --- | --- |
| S(1)-C(12) 1.8065(19)  S(1)-C(13) 1.836(2)  O(1)-C(7) 1.229(2)  O(2)-C(14) 1.215(2)  O(3)-C(24) 1.360(2)  O(3)-C(25) 1.420(3)  O(4)-C(26) 1.380(2)  O(4)-C(27) 1.424(3)  O(5)-C(28) 1.361(2)  O(5)-C(29) 1.426(3)  N(1)-C(7) 1.354(2)  N(1)-C(6) 1.410(2)  N(2)-C(13) 1.456(2)  N(2)-C(11) 1.484(2)  N(2)-C(8) 1.490(2)  N(3)-C(15) 1.314(2)  N(3)-C(16) 1.381(2)  N(4)-C(15) 1.371(2)  N(4)-C(21) 1.380(2) | C(12)-S(1)-C(13) 86.73(8)  C(24)-O(3)-C(25) 116.26(16)  C(26)-O(4)-C(27) 113.33(16)  C(28)-O(5)-C(29) 117.66(17)  C(7)-N(1)-C(6) 111.12(15)  C(7)-N(1)-H(1) 121.8(17)  C(6)-N(1)-H(1) 125.7(17)  C(13)-N(2)-C(11) 110.55(14)  C(13)-N(2)-C(8) 117.56(14)  C(11)-N(2)-C(8) 110.43(13)  C(15)-N(3)-C(16) 104.75(15)  C(15)-N(4)-C(21) 106.16(15)  C(15)-N(4)-H(4) 128.7(17)  C(21)-N(4)-H(4) 124.9(17)  C(2)-C(1)-C(6) 119.37(17)  C(2)-C(1)-C(8) 131.97(17)  C(6)-C(1)-C(8) 108.60(15)  C(1)-C(2)-C(3) 118.65(18)  C(1)-C(2)-H(2) 120.7 |
| Symmetry transformations used to generate equivalent atoms: #1 -x+1,y,-z+1/2 | |

Table S4: Hydrogen bonds for 6i [Å and °].

| D-H...A d(D-H) d(H...A) d(D...A) <(DHA) |
| --- |
| C(11)-H(11)...N(4)#2 1.00 2.70 3.445(2) 131.8  C(20)-H(20)...O(1)#3 0.95 2.52 3.168(2) 125.3  C(25)-H(25C)...N(4)#4 0.98 2.54 3.482(3) 160.1  C(29)-H(29A)...Cl(1^a) 0.98 2.69 3.480(5) 138.4  C(29)-H(29B)...O(5)#5 0.98 2.56 3.372(3) 139.9  C(30)-H(30)...S(1)#6 0.95 2.88 3.819(2) 172.1  N(1)-H(1)...O(1)#7 0.89(3) 2.05(3) 2.934(2) 171(2)  N(4)-H(4)...O(1)#3 0.85(3) 2.41(3) 3.030(2) 131(2)  N(4)-H(4)...N(2)#3 0.85(3) 2.58(3) 3.352(2) 152(2) |
| Symmetry transformations used to generate equivalent atoms:  #1 -x+1,y,-z+1/2 #2 x,-y+1,z+1/2 #3 x,-y+1,z-1/2 #4 -x+1/2,-y+1/2,-z+1 #5 -x+1,-y,-z+1 #6 -x+1,-y+1,-z+1 #7 -x+1/2,-y+3/2,-z+1 |

*Conceptual DFT analysis of the reagents*

The reactivity indices defined within the Conceptual DFT^5,6^ (CDFT) have shown to be powerful tools to understand the reactivity in polar reactions.^7^ The B3LYP/6-31G(d) global reactivity indices, namely, the electronic chemical potential *μ*, chemical hardness *η*, electrophilicity ω and nucleophilicity *N* indices, for AY **7a** and ethylene **3a** are gathered in Table S5.

**Table S5**. B3LYP/6-31G(d) electronic chemical potential *μ*, chemical hardness *η*, electrophilicity ω and nucleophilicity *N* indices, in eV, of AY **7a** and ethylene **3a**.

|  | *μ* | *η* | ω | *N* |
| --- | --- | --- | --- | --- |
| Ethylene **3a** | -4.30 | 3.86 | 2.39 | 2.89 |
| AY **7a** | -3.11 | 3.25 | 1.48 | 4.39 |

The electronic chemical potentia^8^ *μ* of AY **7a**, *μ* = −3.11 eV, is above of that of ethylene **3a**, *μ* = −4.30 eV, indicating that along a polar 32CA reaction the global electron density transfer^9^ (GEDT) will take place from AY **7a** to the ethylene **3a**, the reaction being classified as the forward electron density flux (FEDF).^10,11^

AY **7a** presents an electrophilicity ω index^12^ of 1.48 eV, being classified as a moderate electrophile within the electrophilicity scale,^6^ and a nucleophilicity *N* index^13^ of 4.39 eV, being classified as a strong nucleophile within the electrophilicity scale.^6^ The very strong nucleophilic character of AY **7a**, higher than 4.0 eV, allows its classification as a supernucleophile.^7^

Ethylene **3a** presents an electrophilicity ω index of 2.39 eV, being classified as a strong electrophile within the electrophilicity scale. On the other hand, **3a** presents a nucleophilicity *N* index of 2.89, being also classified as moderate nucleophiles within the nucleophilicity scale.

The supernucleophilic character of AY **7a** together with the strong electrophilic character of ethylene **3a** suggest that the corresponding 32CA reaction will have a high polar character, being classified as FEDF. ^10,11^

Along a polar reaction involving non-symmetric species, the most favorable reaction path involves the two-center interaction between the most electrophilic and the most nucleophilic centers.^14^ Many studies have shown that the analysis of the electrophilic P_k_^+^ and nucleophilic P_k_^-^ Parr functions,^15^ resulting from the excess of spin electron density gathered *via* the GEDT^9^ is one of the most accurate and insightful tools for the analysis of the local reactivity in polar and ionic processes. Hence, according to the characteristics of the reagents, the nucleophilic P_k_^-^ Parr functions of AY **7a**, and the electrophilic P_k_^+^ Parr functions of ethylene **3a** were analyzed (see Figure S1).

**Figure S1**. B3LYP/6-31G(d) nucleophilic P_k_^-^ Parr functions of AY **7a**, and electrophilic P_k_^+^ Parr functions of ethylene **3a**.

The two C1 and C3 carbon of AY **7a** are nucleophilically activated by P_k_^-^ = 0.36 and 0.32, respectively; the C1 carbon being slightly more activated than the C3 carbon. Note that the nitrogen atom is deactivated. On the other hand, the β-conjugated C4 carbon of ethylene **3a** is the most electrophilically activated center, P_k_^+^ = 0.24, of this species. Note that it is twice electrophilically activated than the carbonyl C6 carbon, P_k_^+^ = 0.12. Thus, it is expected that the most favorable two center interactions at the TS will take place between the β-conjugated C4 carbon of ethylene **2** and the C1 or C3 carbon of AY **7a**.

*AIM topological analysis of the electronic structure of the most favorable* ***TS-on***

Finally, the nature of the C3−C4 and C1−C5, and the O−H hydrogen bond (HB) electronic interactions at **TS-on** was studied by an Atom-In-Molecules^16^ (AIM) topological analysis of the electron density *ρ* at the (3,-1) critical points (CPs). The contour line map of Laplacian ∇^2^(r) of the electron density is shown in Figure S2, while the calculated AIM parameters are given in Table S6.

At **TS-on**, the CPs **CP1** and **CP2**, associated with the C3−C4 and C1−C5 interacting regions, respectively, show positive Laplacian ∇^2^(r) values indicating the absence of any covalent interaction (see Table S6). The electron density at **CP1**, 0.0655 e, is three time than that at **CP2**, 0.0226 e, indicating a higher electronic interaction at the C3−C5 region than at the C1−C5 one, in agreement with the geometrical asynchronicity found at the **TS-on** (see Figure S2).

**Figure S2.** Representations of the contour line map of Laplacian ∇^2^(r) of the electron density in the plain of the C3−C4 and C1−C5 interacting regions at **TS-on**. The critical points **CP1** and **CP2** are given in blue, and those associated with O−H HBs, **CP3** and **CP4**, are given in red.

**Table S6.** AIM parameters, in au, of (3,-1) CPs at **TS-on** in the regions associated with the C−C single bond formation, **CP1** and **CP2**, and in the regions associated with the O−H HBs, **CP3** and **CP4**.

| **Type (3,-1)** | **CP1** | **CP2** | **CP3** | **CP4** |
| --- | --- | --- | --- | --- |
| Density ρ(r) | 0.0655 | 0.0226 | 0.0111 | 0.0091 |
| Laplacian ∇^2^(r) | 0.0336 | 0.0466 | 0.0385 | 0.0294 |
| G(r) | 0.0253 | 0.0113 | 0.0084 | 0.0064 |
| K(r) | 0.0169 | 0.0004 | 0.0013 | 0.0009 |
| V(r) | -0.0422 | -0.0108 | -0.0071 | -0.0056 |
| \|V(r)\|/G(r) | 1.6686 | 0.9639 | 0.8497 | 0.8611 |

Espinosa^17^ proposed a useful criterion to characterize interactions at the CPs, the ratio of potential and kinetic energy electron density, |V(r)|/G(r). For ionic, non-covalent and HB interactions, |V(r)|/G(r) < 1. Non-covalent interactions with somewhat covalent character is characterized by 1 < |V(r)|/G(r) < 2, while covalent interactions show |V(r)|/G(r) > 2. The calculated values of |V(r)|/G(r) at the selected CPs are given in Table S6. The |V(r)|/G(r) values of **CP3** and **CP4** are lower than 0.86, indicating that they are associated with HB interactions.

**Table S7.** ωB97XD/6-311G(d,p) total electronic energies (E, in a.u.), enthalpies (H, in a.u.), entropies (S, in cal·mol^-1^K^-1^) and Gibbs free energies (G, in a.u.), computed at 65 ºC in methanol, of the stationary points involved in the 32CA reaction of AY **7a** with ethylene **3a**.

|  | E | H | S | G |
| --- | --- | --- | --- | --- |
| AY **7a** | -1008.046569 | -1007.835329 | 116.688 | -1007.898145 |
| ethylene **3a** | -801.586939 | -801.323495 | 128.119 | -801.392464 |
| **MC-on** | -1809.666536 | -1809.188433 | 202.825 | -1809.297617 |
| **MC-mn** | -1809.660799 | -1809.182977 | 206.888 | -1809.294349 |
| **TS-on** | -1809.654755 | -1809.177514 | 191.94 | -1809.280839 |
| **TS-ox** | -1809.645759 | -1809.168506 | 194.70 | -1809.273317 |
| **TS-mn** | -1809.647504 | -1809.170359 | 196.164 | -1809.275958 |
| **TS-mx** | -1809.642687 | -1809.165763 | 198.282 | -1809.272502 |
| **6a** | -1809.715874 | -1809.235157 | 196.179 | -1809.340764 |
| **8a** | -1809.709871 | -1809.229331 | 197.520 | -1809.335660 |
| **9a** | -1809.711578 | -1809.23066 | 196.675 | -1809.336534 |
| **10a** | -1809.716634 | -1809.235762 | 196.291 | -1809.341429 |

**Biological evaluation**

**NCI screening**

The compound has been processed according to standard method NCI-60 Human Tumor Cell Lines Screen for organic compound at development therapeutic program (DTP). Requests for access to this national resource can be submitted online at:

<https://dtp.cancer.gov/discovery_development/nci-60/default.htm>

**Table S8:** NCI anticancer screening expressed as percentage growth inhibition (GI %) of *in vitro* subpanel tumor cell lines (leukemia, lung, colon, CNS, melanoma, ovary, kidney, prostate, and breast cancers) at 10 μM concentration for the newly synthesized spirooxindole derivative.

| **Subpanel cancer cell Lines** | % Growth Inhibition (GI %) ^a^ | | | | | | | | | | | | | |
| --- | --- | --- | --- | --- | --- | --- | --- | --- | --- | --- | --- | --- | --- | --- |
|  | **6a** | **6b** | **6c** | **6d** | **6e** | **6f** | **6g** | **6h** | **6i** | **6j** | **6k** | **6l** | **6m** | **6n** |
| **Leukemia**  **CCRF-CEM** | 110.88 | 86.28 | 95.01 | 89.52 | 104.05 | 98.19 | 60.10 | 108.18 | 104.28 | 100.83 | 99.91 | 88.15 | 112.16 | 110.43 |
| **HL-60(TB)** | 101.42 | 98.33 | 107.92 | 96.22 | 94.13 | 93.12 | 80.72 | 104.02 | 102.67 | 102.53 | 99.45 | 89.88 | 98.44 | 98.36 |
| **K-562** | 105.35 | 82.33 | 83.09 | 81.57 | 100.16 | 87.36 | 65.60 | 92.49 | 94.21 | 96.37 | 92.18 | 74.53 | 84.44 | 89.83 |
| **MOLT-4** | 100.41 | 68.88 | 82.67 | 78.52 | 96.14 | 83.56 | 43.04 | 91.20 | 97.36 | 89.52 | 85.61 | 56.27 | 85.55 | 96.84 |
| **RPMI-8226** | 110.61 | 63.19 | 72.01 | 80.28 | 100.55 | 87.75 | 42.49 | 82.44 | 107.80 | 103.88 | -------- | 61.34 | 83.61 | 100.76 |
| **SR** | 94.18 | 65.87 | 85.62 | 102.39 | 100.77 | 81.31 | 45.80 | 79.14 | 91.76 | 93.99 | 84.27 | 54.14 | 82.78 | 86.83 |
| **Non-Small Cell Lung Cancer**  **A549/ATCC** | 98.71 | 81.41 | 97.12 | 75.39 | 87.10 | 78.56 | 61.99 | 95.44 | 89.42 | 98.66 | 94.16 | 73.00 | 84.63 | 101.52 |
| **EKVX** | 105.85 | 80.73 | 86.98 | 71.63 | 100.29 | 93.83 | 63.51 | 96.61 | 100.34 | 91.60 | 91.36 | 68.85 | 88.18 | 103.06 |
| **HOP-62** | 99.29 | 91.65 | 89.53 | 104.89 | 99.51 | 104.18 | 80.29 | 96.42 | 104.22 | 85.25 | 95.74 | 88.73 | 97.91 | 99.77 |
| **HOP-92** | 95.55 | 100.35 | 108.61 | 87.02 | 105.13 | 112.43 | 77.60 | 98.35 | 106.27 | 97.35 | 103.67 | 96.93 | 99.65 | 112.91 |
| **NCI-H226** | 95.35 | 74.00 | 78.96 | 73.86 | -------- | -------- | 61.76 | 99.13 | -------- | 91.26 | 91.36 | 74.82 | 92.57 | 92.20 |
| **NCI-H23** | 104.15 | 81.40 | 82.32 | 91.15 | 103.32 | 97.97 | 85.36 | 99.35 | 103.53 | 96.45 | 94.29 | 75.05 | 90.65 | 92.93 |
| **NCI-H322M** | 98.07 | 93.51 | 91.13 | 83.06 | 97.73 | 90.42 | 79.48 | 93.50 | 93.11 | 99.79 | 99.83 | 89.91 | 99.75 | 101.36 |
| **NCI-H460** | 106.16 | 74.31 | 66.84 | 84.49 | 101.47 | 88.87 | 55.51 | 94.83 | 104.98 | 97.40 | 101.76 | 66.20 | 97.26 | 102.09 |
| **NCI-H522** | 93.69 | 87.91 | 87.67 | 84.34 | 91.46 | 79.48 | 71.68 | 83.86 | 88.26 | 87.18 | 87.93 | 91.16 | 77.81 | 99.69 |
| **Colon Cancer**  **COLO 205** | 99.94 | -------- | -------- | 81.92 | 111.99 | 109.03 | -------- | -------- | 114.23 | -------- | -------- | -------- | -------- | -------- |
| **HCC-2998** | 109.49 | 92.13 | 99.64 | 94.89 | 108.42 | 96.95 | 70.31 | 101.14 | 107.65 | 102.58 | 102.48 | 79.42 | 91.78 | 103.97 |
| **HCT-116** | 105.91 | 60.50 | 60.00 | 74.49 | 96.32 | 92.93 | 56.41 | 88.95 | 102.49 | 96.27 | 101.52 | 59.19 | 84.40 | 98.62 |
| **HCT-15** | 108.59 | 76.58 | 88.88 | 83.59 | 100.07 | 93.62 | 55.97 | 102.73 | 105.06 | 101.16 | 99.07 | 67.04 | 95.02 | 104.68 |
| **HT29** | 98.83 | 80.93 | 74.73 | 69.17 | 101.00 | 85.65 | 61.90 | 103.57 | 107.18 | 99.33 | 99.27 | 80.24 | 86.03 | 115.18 |
| **KM12** | 104.70 | 66.55 | 71.84 | 75.44 | 98.81 | 88.74 | 52.28 | 89.43 | 101.19 | 82.49 | 95.67 | 59.84 | 98.10 | 101.66 |
| **SW-620** | 97.80 | 77.05 | 84.13 | 92.05 | 96.71 | 91.13 | 72.92 | 91.84 | 99.50 | 95.36 | 100.18 | 80.73 | 89.14 | 95.60 |
| **CNS Cancer**  **SF-268** | 98.13 | 77.50 | 90.19 | 63.98 | 92.37 | 96.28 | 47.05 | 88.50 | 99.25 | 91.72 | 92.12 | 71.47 | 88.57 | 101.51 |
| **SF-295** | 104.34 | 77.48 | 95.33 | 58.97 | 92.61 | 77.94 | 63.69 | 105.07 | 95.22 | 96.29 | 103.20 | 63.87 | 96.14 | 109.43 |
| **SF-539** | 95.10 | 84.14 | 91.62 | 81.92 | 101.06 | 97.71 | 76.42 | 90.03 | 99.58 | 98.50 | 99.62 | 82.40 | 86.74 | 96.44 |
| **SNB-19** | 96.98 | 77.66 | 84.90 | 80.28 | 103.91 | 95.87 | 70.06 | 97.76 | 100.12 | 93.65 | 91.66 | 66.97 | 91.94 | 96.82 |
| **SNB-75** | 79.85 | 69.13 | 72.88 | 61.35 | 85.89 | 89.83 | 59.67 | 74.48 | 94.06 | 73.14 | 88.31 | 68.04 | 77.74 | 95.95 |
| **U251** | 93.91 | 81.17 | 93.23 | 73.26 | 100.39 | 93.48 | 79.03 | 98.92 | 102.92 | 95.82 | 102.89 | 75.79 | 105.82 | 108.54 |
| **Melanoma**  **LOX IMVI** | 98.70 | 76.59 | 88.71 | 82.02 | 97.53 | 94.75 | 67.22 | 84.30 | 101.07 | 89.25 | 94.22 | 73.15 | 87.50 | 91.70 |
| **MALME-3M** | 100.62 | 89.65 | 94.89 | 85.35 | 100.83 | 94.21 | 86.19 | 96.83 | 99.79 | 105.00 | 94.65 | 78.55 | 97.55 | 101.71 |
| **M14** | 107.26 | 79.39 | 83.34 | 74.73 | 100.48 | 94.55 | 66.76 | 97.34 | 107.01 | 95.11 | 103.11 | 68.64 | 96.97 | 101.05 |
| **MDA-MB-435** | 102.53 | 79.86 | 88.35 | 70.57 | 100.43 | 94.59 | 56.02 | 93.48 | 100.86 | 92.74 | 96.84 | 67.86 | 96.48 | 105.39 |
| **SK-MEL-2** | 100.65 | 110.21 | 108.47 | 93.60 | 110.84 | 102.33 | 84.40 | 102.41 | 114.67 | 103.59 | 105.74 | 107.19 | 95.69 | 124.11 |
| **SK-MEL-28** | 102.99 | 92.15 | 97.72 | 98.79 | 105.02 | 101.66 | 78.32 | 95.27 | 106.84 | 98.97 | 96.12 | 84.83 | 101.70 | 106.54 |
| **SK-MEL-5** | 92.69 | 91.56 | 94.19 | 83.29 | 99.05 | 94.71 | 81.86 | 93.74 | 103.31 | 94.04 | 98.17 | 84.85 | 88.78 | 98.16 |
| **UACC-257** | 97.28 | 95.17 | 98.52 | 80.56 | 93.36 | 83.86 | 74.41 | 91.90 | 87.78 | 95.60 | 90.20 | 77.87 | 99.05 | 115.71 |
| **UACC-62** | 95.72 | 73.87 | 77.52 | 77.95 | 90.84 | 78.07 | 57.04 | 80.01 | 87.42 | 77.28 | 84.40 | 70.51 | 80.08 | 90.58 |
| **Ovarian Cancer**  **IGROV1** | 91.43 | 68.93 | 67.79 | 74.98 | 89.67 | 93.15 | 54.84 | 87.88 | 95.85 | 92.11 | 89.82 | 63.65 | 84.60 | 92.37 |
| **OVCAR-3** | 110.04 | 86.41 | 87.71 | 88.96 | 104.60 | 100.84 | 69.66 | 97.23 | 109.20 | 96.26 | 102.79 | 79.77 | 103.04 | 109.69 |
| **OVCAR-4** | 108.12 | 69.50 | 80.93 | 62.36 | 93.92 | 83.21 | 57.64 | 82.07 | 100.25 | 86.55 | 91.48 | 70.79 | 87.28 | 99.23 |
| **OVCAR-5** | 104.20 | 92.55 | 96.13 | 95.15 | 96.16 | 100.38 | 86.07 | 92.88 | 94.26 | 101.91 | 95.59 | 95.86 | 90.53 | 97.16 |
| **OVCAR-8** | 96.14 | 89.54 | 98.85 | 95.91 | 96.75 | 94.69 | 76.96 | 97.05 | 99.27 | 97.69 | 105.27 | 89.75 | 94.86 | 100.74 |
| **NCI/ADR-RES** | 104.53 | 84.65 | 85.97 | 86.22 | 102.34 | 99.38 | 68.39 | 97.07 | 104.25 | 95.86 | 96.04 | 83.21 | 95.89 | 100.68 |
| **SK-OV-3** | 99.14 | -------- | -------- | 94.17 | 101.06 | 101.74 | -------- | -------- | 102.46 | -------- | -------- | -------- | -------- | -------- |
| **Renal Cancer**  **786-0** | 102.33 | 97.58 | 99.39 | 86.12 | 100.38 | 99.04 | 85.90 | 100.74 | 99.42 | 106.36 | 103.34 | 91.52 | 99.96 | 103.99 |
| **A498** | -------- | -------- | -------- | -------- | 79.70 | 57.17 | -------- | -------- | 81.66 | -------- | -------- | -------- | -------- | -------- |
| **ACHN** | 99.52 | 78.22 | 89.63 | 88.90 | 102.71 | 94.19 | 63.69 | 92.78 | 106.70 | 92.21 | 99.02 | 74.58 | 90.18 | 93.67 |
| **CAKI-1** | 86.02 | 68.30 | 78.60 | 54.49 | 89.62 | 90.02 | 36.91 | 84.09 | 92.56 | 84.05 | 85.78 | 60.27 | 74.67 | 88.49 |
| **RXF 393** | 101.44 | 71.11 | 90.14 | 57.11 | 111.88 | 99.62 | 40.70 | 104.04 | 109.21 | 88.24 | 99.72 | 62.12 | 85.24 | 104.00 |
| **SN12C** | 105.63 | 80.68 | 90.73 | 85.23 | 97.48 | 91.09 | 62.97 | 94.77 | 99.02 | 94.32 | 93.96 | 82.75 | 96.07 | 92.51 |
| **TK-10** | 109.81 | 120.78 | 120.19 | 94.45 | 105.73 | 101.30 | 107.36 | 112.64 | 103.54 | 115.18 | 110.78 | 145.44 | 116.30 | 132.59 |
| **UO-31** | 77.20 | 49.05 | 55.37 | 54.29 | 76.00 | 77.04 | 39.72 | 60.63 | 86.62 | 73.89 | 73.38 | 48.62 | 65.34 | 61.68 |
| **DU-145** | 114.91 | 97.36 | 97.02 | 105.68 | 108.80 | 111.30 | 86.89 | 105.49 | 111.22 | 98.67 | 104.12 | 97.13 | 104.79 | 113.52 |
| **Breast Cancer**  **MCF7** | 98.09 | 74.65 | 77.73 | 70.38 | 94.54 | 87.42 | 51.05 | 92.83 | 99.86 | 92.41 | 93.63 | 60.57 | 91.17 | 100.10 |
| **HS 578T** | 102.63 | 72.07 | 83.87 | 78.62 | 100.94 | 98.05 | 65.14 | 80.41 | 101.58 | 84.98 | 91.73 | 67.58 | 78.23 | 91.29 |
| **BT-549** | 101.74 | 96.97 | 107.45 | 96.21 | 94.12 | 97.44 | 92.26 | 106.65 | 93.72 | 93.60 | 107.95 | 90.25 | 99.50 | 109.71 |
| **T-47D** | 88.05 | 59.91 | 69.68 | 68.76 | 90.53 | 84.06 | 54.41 | 76.28 | 101.79 | 80.94 | 82.99 | 54.68 | 79.83 | 84.78 |
| **MDA-MB-468** | 100.18 | 89.44 | 91.60 | 63.53 | 100.24 | 94.20 | 69.15 | 104.00 | 99.64 | 102.05 | 99.84 | 87.17 | 99.30 | 102.35 |

**Determination of cytotoxicity of synthetic compounds to normal human cell line**

Normal human lung fibroblast Wi-38 cell line was used to detect cytotoxicity of the studied compounds (Table S8). Wi-38 cell line was cultured in DMEM medium-contained 10% fetal bovine serum (FBS), seeded as 5x10^3^ cells per well in 96-well cell culture plate and incubated at 37ºC in 5% CO_2_ incubator. After 24 h for cell attachment, serial concentrations of the synthetic compounds were incubated with Wi-38 cells for 72 h. Cell viability was assayed by MTT method ([Mosmann, 1983](#_ENREF_122)). Twenty microliters of 5 mg/ml MTT (Sigma, USA) was added to each well and the plate was incubated at 37 ºC for 3 h. Then MTT solution was removed, 100 µl DMSO was added and the absorbance of each well was measured with a microplate reader (BMG LabTech, Germany) at 570 nm. The effective safe concentration (EC_100_) value (at 100% cell viability) of the tested compounds was estimated by the Graphpad Instat software.

**Supplementary Table S9.** The percentage of Wi-38 viability and the growth inhibition percentages of MDA-MB231 and PC-3 cells after incubation with 5 µM of different tested compounds**.**

| **Chemical Structure** | **Wi-38** | **MDA-MB 231** | **PC-3** |
| --- | --- | --- | --- |
| ****  **6a** | **95.800±0.917** | **55.543±0.761** | **55.313±0.521** |
| ****  **6b** | 49.592**±**1.192 | 52.717**±**2.935 | 51.875**±**0.000 |
| ****  **6c** | 59.858**±**2.842 | 55.543**±**2.500 | 54.063**±**1.354 |
| ****  **6d** | **83.425±0.092** | **51.739±1.304** | **49.896±0.938** |
| ****  **6e** | 51.700**±**1.283 | 58.152**±**1.848 | 58.229**±**0.729 |
| ****  **6f** | 47.392**±**0.092 | 44.891**±**3.587 | 46.042**±**0.833 |
| ****  **6g** | 39.967**±**1.100 | 56.739**±**0.870 | 54.479**±**1.563 |
| ****  **6h** | 50.967**±**1.833 | 40.217**±**0.000 | 40.417**±**1.250 |
| ****  **6i** | 43.267**±**0.917 | 45.978**±**1.413 | 49.792**±**3.125 |
| ****  **6j** | 39.967**±**2.383 | 40.761**±**1.630 | 40.313**±**3.437 |
| ****  **6k** | 38.592**±**3.025 | 37.609**±**2.609 | 41.563**±**0.729 |
| ****  **6l** | 37.400**±**2.017 | 53.587**±**0.109 | 55.104**±**1.979 |
| ****  **6m** | 43.725**±**2.292 | 62.826**±**3.913 | 60.104**±**0.521 |
| ****  **6n** | 46.200**±**1.467 | 52.935**±**2.500 | 52.500**±**1.458 |

All values are presented as mean ± SEM.

**Investigation of the cytotoxicity of the studied compounds against cancerous human cell lines**

**MTT assay for determination of the anticancer activity**

Anticancer effect of the above-mentioned compounds was assayed using three human cancer cell lines. Triple negative breast cancer cell line (MDA-MB 231) and prostate cancer cell line (PC3) were cultured in DMEM (Lonza, USA) supplemented with 10% FBS. All cancer cells (4x10^3^ cells/well) were seeded in sterile 96-well plates. After 24h, serial concentrations of the tested compounds were incubated with three cancer cell lines for 72 h at 37 ºC in 5% CO_2_ incubator. MTT method was done as described above. The half maximal inhibitory concentration (IC_50_) values were calculated using the Graphpad Instat software. Furthermore, cellular morphological changes before and after treatment with the most effective and safest anticancer compounds were investigated using phase contrast inverted microscope with a digital camera (Olympus, Japan).

**Flow cytometric analysis of apoptotic effect of the most effective anticancer compounds**

The most active compounds were incubated, for 72 h, with MDA-MB231 and PC3 cell lines. After trypsinization, the untreated and treated cells were incubated with annexin V/PI for 15 min. Then cells were fixed and incubated with streptavidin-fluorescein (5 µg/mL) for 15 min. The apoptosis-dependent anticancer effect was determined by quantification of annexin-stained apoptotic cells using the FITC signal detector (FL1) against the phycoerythrin emission signal detector (FL2).

**Quantitative detection for the change in the expression of p21, cyclin-D and nuclear factor-kappa B (NF-**κ**B) genes in the treated cancer cells**

Total RNAs of untreated and the two most effective anticancer compounds-treated MDA-MB 231 cells were extracted using Gene JET RNA Purification Kit (Thermo Scientific, USA). The cDNA was synthesized from mRNA using cDNA Synthesis Kit (Thermo Scientific, USA). Real time PCR was performed using SYBR green master mix and specific primers (Forward/Reverse) were 5′-CTGGGGATGTCCGTCAGAAC-3′/5′-GCCATTAGCGCATCACAGT-3′, 5′-GCGGAG GAGAACAAACAGAT-3′/5′-TGAACTTCACATCTGTGGCA-3′ and 5′-TACTCTGGCGCAGAAATTAGGTC-3′/5′-CTGTCTCGGAGCTCGTCTATTTG-3′ for p21, cyclin D and NF-κB genes, respectively. The 2^−ΔΔCT^ equation was used to estimate the change in gene expressions in the treated cancer cells relative to untreated cancer cells.

**Immunohistochemical detection of proliferation marker (Ki-67) and tumor suppressor protein (p53)**

After trypsinization, the untreated and treated MDA-MB231 cells were centrifuged and washed with PBS buffer and then 10% formalin in PBS was added to cell pellets. The fixed cell specimens were dehydrated in ascending grades of alcohol and immersed in xylene for one hour (three times) followed by impregnation in melted paraffin to from solid paraffin blocks. Then a rotator microtome was used to cut each block into 3-5 μm thick sections that were transferred into positively charge slides. Slides were dried at 60-70°C for 1-2 h then dewaxed by immersion in xylene 3 times and rehydrated in descending grading ethanol. After that slides were incubated in 3% H_2_O_2_ for 10 min, washed in PBS buffer twice for 3 min and put in 10 mM citrate buffer (pH) followed by heating for 10-20 min. After cooling and washing in PBS, slides were separately soaked overnight in primary antibodies (anti-Ki-67 and anti-p53). Slides were washed in PBS, covered with biotinylated goat anti-polyvalent secondary antibody for 10 min and then streptavidin peroxidase was added. After 10 min, substrate of secondary antibody (3,3'-diaminobenzidine) was added followed by washing in PBS and placing in hematoxylin bath for 1-4 min then washing in PBS (1 min) and water (3 min). Percentage of immunostained cells was evaluated by imaging analysis cellSens software of phase contrast microscope (Olympus, Japan).

**Determination of p53 activation in the treated cancer cells relative to the untreated cells**

The relative fold activation of p53 in the treated MDA-MB 231 cells were quantified using human p53 transcription factor activity assay kit (RayBio, USA). Briefly, 100 µl of lysates of the untreated and treated MDA-MB 231 cells were added to p53 probe-coated 96 wells and incubated for 2 h (these oligonucleotides capture the active p53 in cell lysate). After washing for discarding unbound p53, primary antibody was added to bound p53-DNA complex, incubated for 1 h. Each well was washed then peroxidase-conjugated secondary antibody was added and incubated for 1 h. After washing and incubating with substrate for 30 min, the absorbance was measured at 450 nm.

**Statistical analysis**

The data are expressed as mean ± standard error of mean (SEM) and the significant values were considered at *p* < 0.05. One-way analysis of variance (ANOVA) by Tukey’s test used for evaluating the difference between the mean values of the studied treatments. The analysis was done for three measurements using SPSS software version 16.

**MDM2** **binding analysis by microscale thermophoresis (MST) assay**

MST is a complementary biophysical assay to assess the binding of a small molecule to its receptor. For example, MST (NanoTemper Technologies GmbH) was used to determine the binding affinities between Mdm2 (residues 1–118 T47W; 500 nM) and inhibitors. T47W mutation was introduced to facilitate label-free measurements and did not interfere with small molecule binding, as evidenced by unaffected affinity toward a reference compound, nutlin-3. Experiments were performed in 50 mM phosphate buffer at pH 7.4 containing 150 mM NaCl, 5 mM DTT, and 5% DMSO. Inhibitors at increasing concentration (0.763 nM to 25 μM; highest concentration was limited by solubility) were incubated with the protein for 5 min prior to measurement at 25 °C (excitation 280 nm, emission 350 nm). An inhibitor concentration-dependent decrease in tryptophan fluorescence was observed. Inhibitor binding-specific fluorescence quenching was evidenced by a loss of the effect in samples containing the inhibitor, but denatured by heating (95 °C for 5 min) in the presence of 2% SDS and 20 mM DTT. Kd values and uncertainties were calculated using the MO Affinity Analysis software.

**References**

1. Rikagu Oxford Diffraction, *CrysAlisPro*, 2020, Rikagu Oxford Diffraction inc., Yarnton, Oxfordshire, England.
2. Sheldrick, G. M. *Acta Cryst.* **2015**, *A71*, 3-8.
3. Sheldrick, G. M. *Acta Cryst.* **2015**, *C71*, 3-8.
4. Hübschle, C. B.; Sheldrick, G. M.; Dittrich, B. *J. Appl. Cryst.* **2011**, *44*, 1281-1284.
5. Parr, R.G.; Yang, W. *Density Functional Theory of Atoms and Molecules*; Oxford University Press: New York, NY, USA, 1989.
6. Domingo, L.R.; Ríos‐Gutiérrez, M.; Pérez, P. Applications of the conceptual density functional indices to organic chemistry reactivity. *Molecules* **2016**, *21*, 748.
7. Domingo, L.R.; Ríos-Gutiérrez, M. *Application of Reactivity Indices in the Study of Polar Diels–Alder Reactions in Conceptual Density Functional Theory: Towards a New Chemical Reactivity Theory*; Liu, S., Ed.; WILEY-VCH GmbH: Weinheim, German, 2022; Volume 2, pp. 481–502.
8. Parr, R.G.; Pearson, R.G. Absolute hardness: Companion parameter to absolute electronegativity. *J. Am. Chem. Soc.* **1983**, *105*, 7512–7516.
9. Domingo, L.R. A new C–C bond formation model based on the quantum chemical topology of electron density. RSC Adv. **2014**, 4, 32415–32428.
10. Domingo, L.R.; Ríos‐Gutiérrez, M.; Pérez, P. A Molecular Electron Density Theory Study of the Reactivity of Tetrazines in Aza‐ Diels‐Alder Reactions. *RSC Adv.* **2020**, *10*, 15394–15405.
11. Domingo, L.R.; Ríos‐Gutiérrez. A Useful Classification of Organic Reactions Bases on the Flux of the Electron Density. *Sci. Rad.* **2023**, *2*, 1.
12. Parr, R.G.; Szentpaly, L.V.; Liu, S. Electrophilicity index. *J. Am. Chem. Soc.* **1999**, *121*, 1922–1924.
13. Domingo, L.R.; Chamorro, E.; Pérez, P. Understanding the reactivity of captodative ethylenes in polar cycloaddition reactions. A theoretical study. *J. Org. Chem.* **2008**, *73*, 4615–4624.
14. Aurell, M.J.; Domingo, L.R.; Perez, P.; Contreras, R. A theoretical study on the regioselectivity of 1,3‐dipolar cycloadditions using DFT‐based reactivity indexes. *Tetrahedron* **2004**, *60*, 11503–11509.
15. Domingo, L.R.; Pérez, P.; Sáez, J.A. Understanding the local reactivity in polar organic reactions through electrophilic and nucleophilic Parr functions. RSC Adv. **2013**, 3, 1486–1494.
16. Bader, R.F.W.; Tang, Y.H.; Tal, Y.; Biegler-König, F.W. Properties of atoms and bonds in hydrocarbon molecules. J. Am. Chem. Soc. 1982, 104, 946–952.
17. Espinosa, E.; Alkorta, I.; Elguero, J.; Molins, E. From weak to strong interactions: A comprehensive analysis of the topological and energetic properties of the electron density distribution involving X–H⋯F–YX–H⋯F–Y systems. *J. Chem. Phys.* **2002**, *117*, 5529–5542.


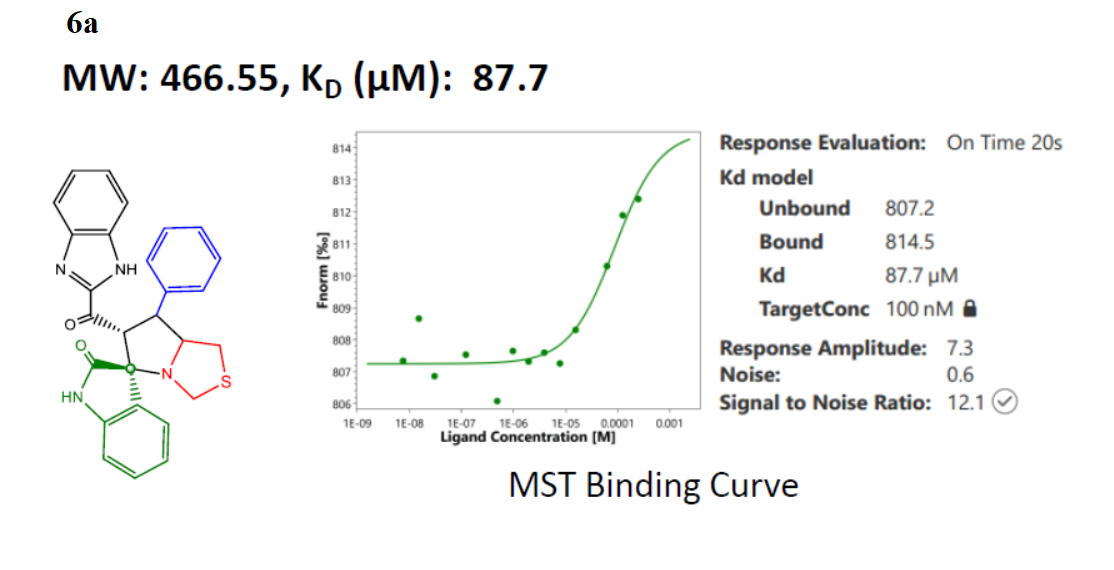


**Figure S3**. MST Binding Curve for compound **6a**


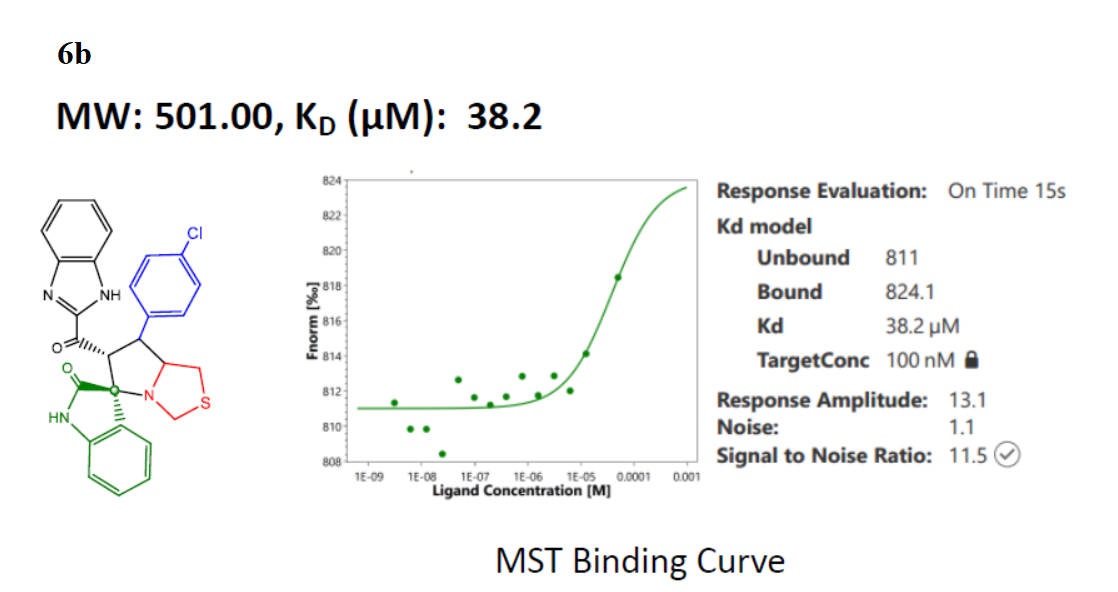


**Figure S4**. MST Binding Curve for compound **6b**


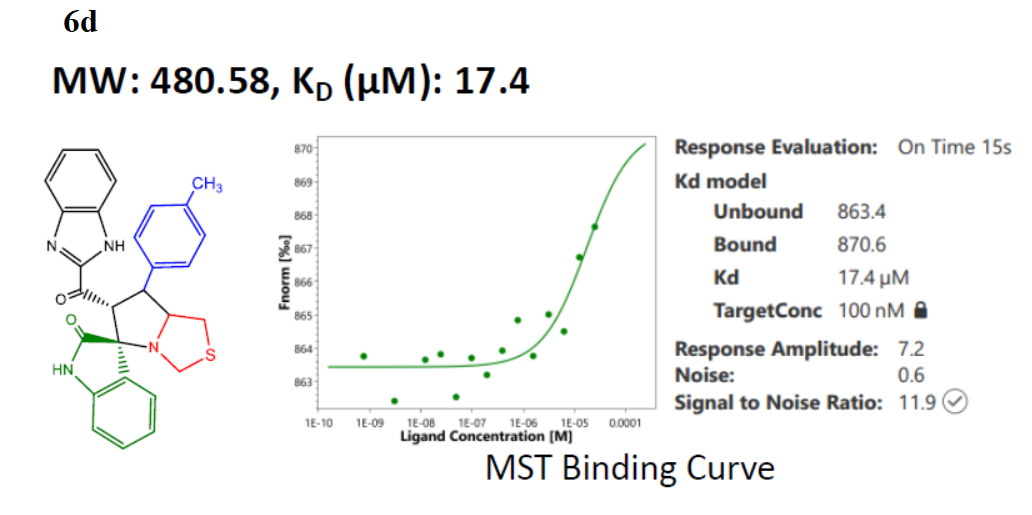


**Figure S5**. MST Binding Curve for compound **6d**


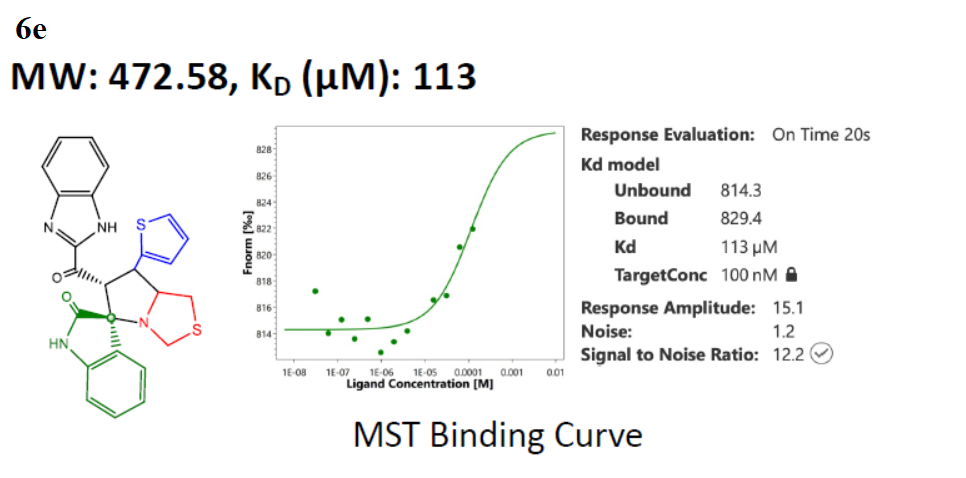


**Figure S6**. MST Binding Curve for compound **6e**


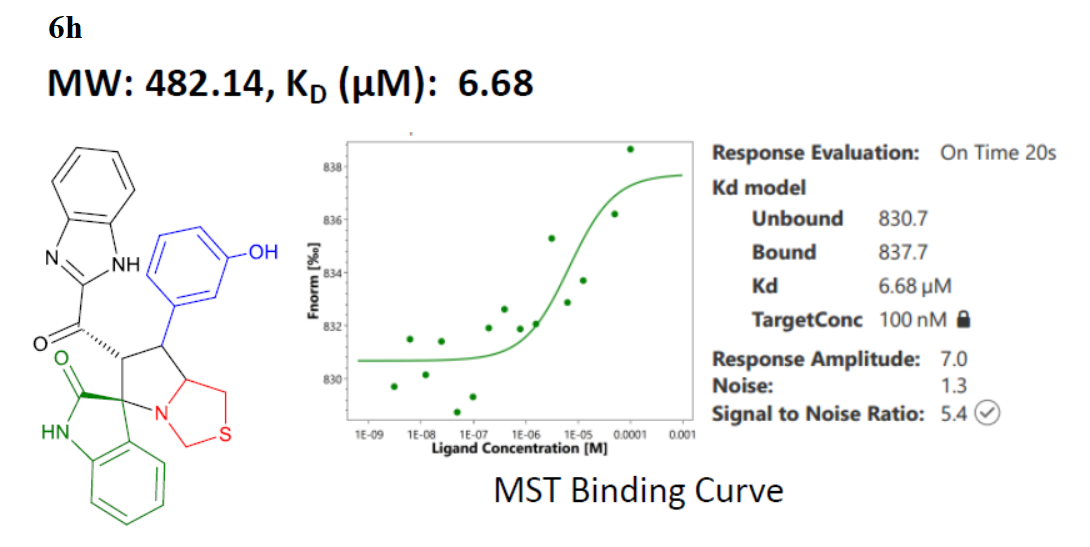


**Figure S7**. MST Binding Curve for compound **6h**


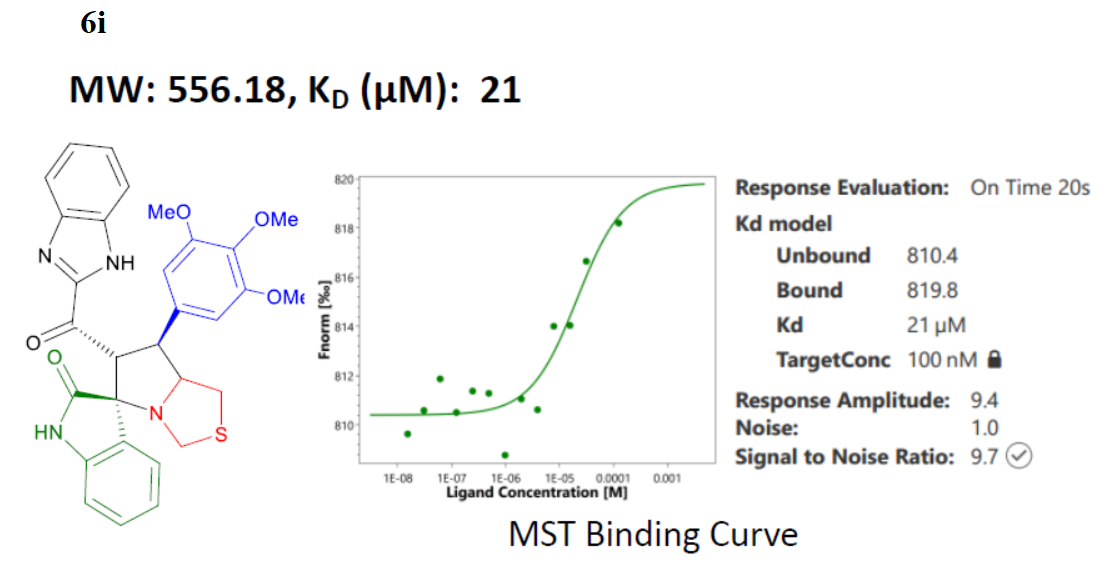


**Figure S8**. MST Binding Curve for compound **6i**


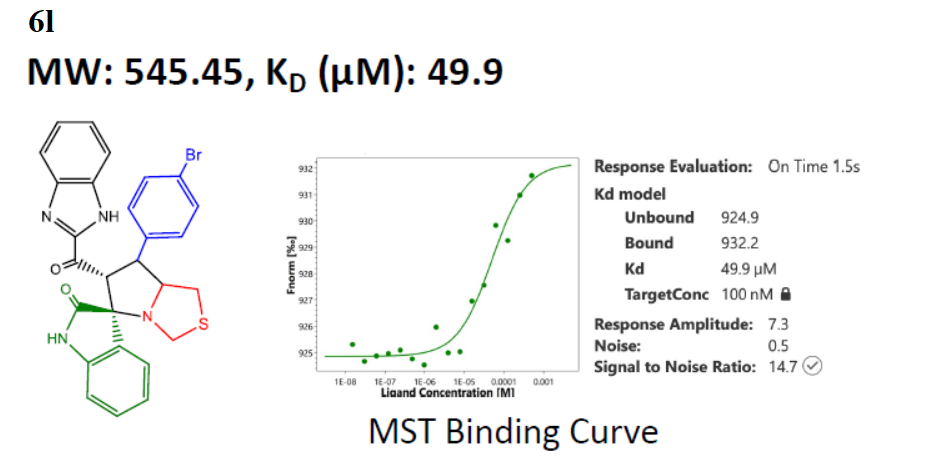


**Figure S9**. MST Binding Curve for compound **6l**


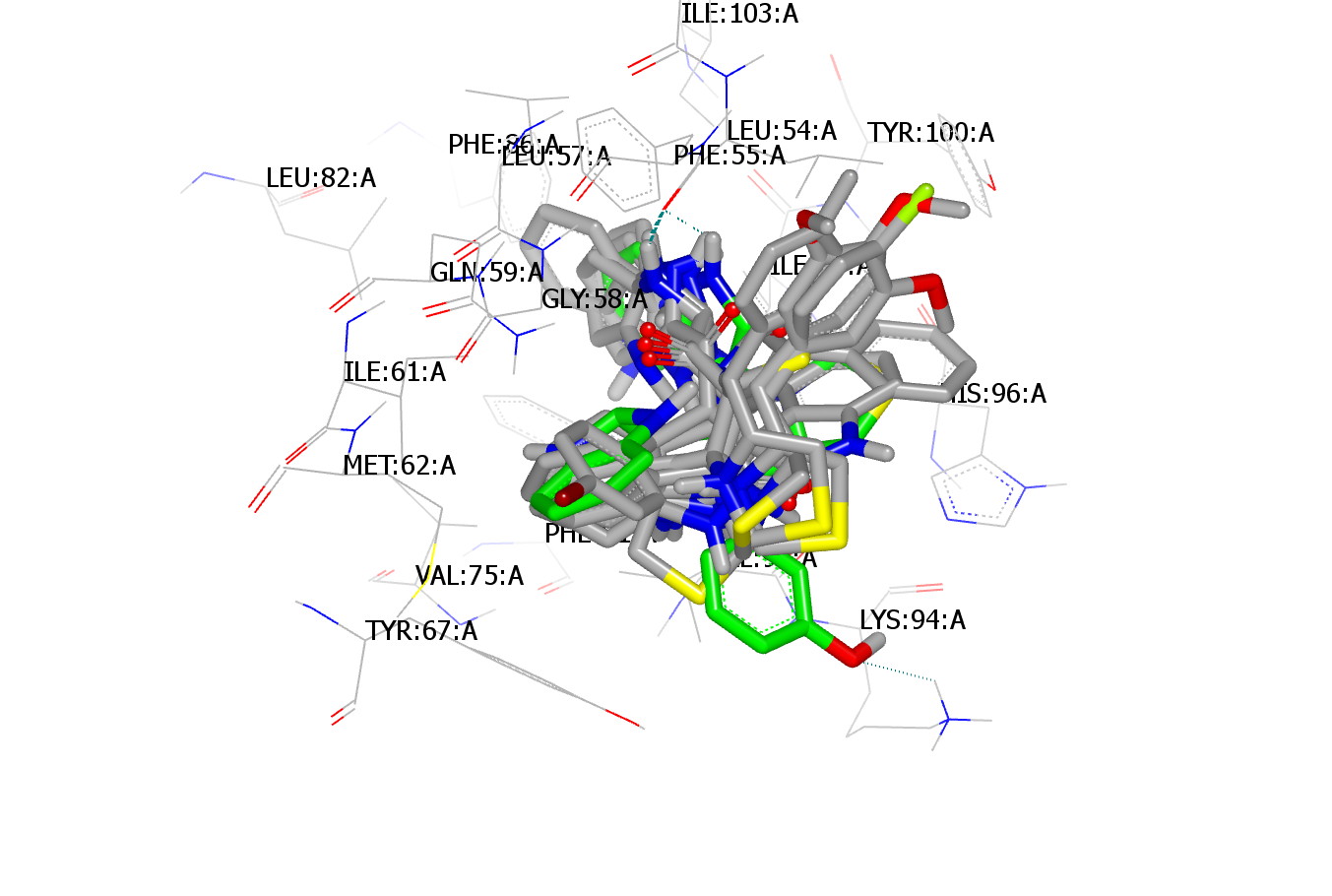


**Figure S10**: Overlay of the synthesized compounds inside the active site of MDM2.

**Figure S11.** ^1^H-NMR and ^13^C-NMR for product **6a**

**Figure S12.** ^1^H-NMR and ^13^C-NMR for product **6b**

**Figure S13.** ^1^H-NMR and ^13^C-NMR for product **6d**

**Figure S14.** ^1^H-NMR and ^13^C-NMR for product **6e**

**Figure S15.** ^1^H-NMR and ^13^C-NMR for product **6f**

**Figure S16.** ^1^H-NMR and ^13^C-NMR for product **6g**

**Figure S17.** ^1^H-NMR and ^13^C-NMR for product **6h**

**Figure S18.** ^1^H-NMR and ^13^C-NMR for product **6i**

**Figure S19.** ^1^H-NMR and ^13^C-NMR for product **6j**

**Figure S20.** ^1^H-NMR and ^13^C-NMR for product **6k**

**Figure S21.** ^1^H-NMR and ^13^C-NMR for product **6l**

**Figure S22.** ^1^H-NMR and ^13^C-NMR for product **6m**

**Figure S23.** ^1^H-NMR and ^13^C-NMR for product **6n**
